# Cell–cell heterogeneity in phosphoenolpyruvate carboxylase biases early cell fate priming in *Dictyostelium discoideum*

**DOI:** 10.3389/fcell.2024.1526795

**Published:** 2025-02-04

**Authors:** Kenichi Abe, Hidenori Hashimura, Haruka Hiraoka, Shoko Fujishiro, Narufumi Kameya, Kazuteru Taoka, Satoshi Kuwana, Masashi Fukuzawa, Satoshi Sawai

**Affiliations:** ^1^ Department of Biological Sciences, Graduate School of Science, The University of Tokyo, Bunkyō, Japan; ^2^ Department of Basic Science, Graduate School of Arts and Sciences, The University of Tokyo, Meguro, Japan; ^3^ Graduate School of Frontier Biosciences, Osaka University, Suita, Japan; ^4^ Department of Biology, Faculty of Agriculture and Life Science, Hirosaki University, Hirosaki, Japan; ^5^ Research Center for Complex Systems Biology, Universal Biology Institute, The University of Tokyo, Meguro, Japan

**Keywords:** PEPC, cell fate, cell metabolism, cell heterogeneity, cell differentiation, *Dictyostelium discoideum*

## Abstract

Glucose metabolism is a key factor characterizing the cellular state during multicellular development. In metazoans, the metabolic state of undifferentiated cells correlates with growth/differentiation transition and cell fate determination. Notably, the cell fate of the Amoebozoa species *Dictyostelium discoideum* is biased by the presence of glucose and is also correlated with early differences in intracellular ATP. However, the relationship between early cell–cell heterogeneity, cell differentiation, and the metabolic state is unclear. To address the link between glucose metabolism and cell differentiation in *D. discoideum*, we studied the role of phosphoenolpyruvate carboxylase (PEPC), a key enzyme in the PEP-oxaloacetate-pyruvate node, a core junction that dictates the metabolic flux of glycolysis, the TCA cycle, and gluconeogenesis. We demonstrate that there is cell–cell heterogeneity in PEPC promoter activity in vegetative cells, which depends on nutrient conditions, and that cells with high PEPC promoter activity differentiate into spores. The PEPC null mutant exhibited an aberrantly high prestalk/prespore ratio, and the spore mass of the fruiting body was glassy and consisted of immature spores. Furthermore, the PEPC null mutant had high ATP levels and low mitochondrial membrane potential. Our results suggest the importance of cell–cell heterogeneity in the levels of metabolic enzymes during early cell fate priming.

## 1 Introduction

Cell fate determination is fundamental for the development of multicellular organisms. Recently, cellular metabolism has received particular attention as a determinant of cellular function and specialization (Kim and DeBerardinis, 2019; Jackson and Finley, 2024). In ES cells, accumulation of α-ketoglutarate and acetyl-CoA, the major constituents of glucose metabolism control cell fate decision by promoting epigenetic changes (Moussaieff et al., 2015; TeSlaa et al., 2016). In developing embryos, metabolic activity gradients during tail bud elongation and gastrulation influence cellular signaling pathways and guide cell migration, and hence, pattern formation (Bulusu et al., 2017; Oginuma et al., 2017; Oginuma et al., 2020; Cao et al., 2024). In plants, quiescent center stemness in the root apical meristem is preserved by specific metabolic activities (Mira et al., 2023). In the shoot apical meristem, reactive oxygen species are the key factors in stem cell maintenance and differentiation (Zeng et al., 2017; Wang S. et al., 2024). There are differences in morphogenesis between plants and animals; however, cellular metabolism is integral to cell fate determination and cellular functions, with genetic regulation during development. These studies suggest a common metabolic basis across species that underlies cell fate determination and symmetry breaking during multicellular development.

Multicellularity has also been studied in the cellular slime mold *Dictyostelids*, including the well-studied model system *Dictyostelium discoideum*. *D. discoideum* cells under starvation aggregate and differentiate into two major cell types, prestalk and prespore cells, which first appear in a ‘salt-and-pepper’ manner in the aggregates. Cells then sort and form the anterior–posterior axis, which gives rise to a migrating slug and, ultimately, a fruiting body. The origin of prestalk and prespore differentiation is still unclear; however, cell–cell heterogeneities during the vegetative stage are strongly correlated with later cell fate. These include the cell cycle position (Weijer et al., 1984; Gomer and Firtel, 1987; Araki et al., 1994; Thompson and Kay, 2000; Gruenheit et al., 2018), intracellular calcium levels (Schaap et al., 1996; Kubohara et al., 2007), and intracellular pH levels (Gross et al., 1983; Kubohara et al., 2007). Furthermore, cells grown in glucose-rich medium when mixed with cells grown without have high probability of differentiating into spores (Leach et al., 1973; Thompson and Kay, 2000). Cells with high ATP levels differentiate into stalks and pharmacological perturbation of glycolysis or mitochondrial activity diminishes stalk bias (Hiraoka et al., 2020). This suggests a close relationship between glucose metabolic enzymes and cell fate bias in *D. discoideum*.

We studied the role of phosphoenolpyruvate carboxylase (PEPC; EC 4.1.1.31) in cell differentiation of *D. discoideum*. PEPC is a core enzyme involved in the irreversible carboxylation of phosphoenolpyruvate to generate oxaloacetate and inorganic phosphate (Pi) (O’Leary et al., 2011). PEPC is a major component of the so-called “PEP-pyruvate-oxaloacetate node” which provides the direct precursors for gluconeogenesis and anaplerosis of TCA cycle (Sauer and Eikmanns, 2005). PEPC is present in the genomes of many bacteria, plants, chytrids and protists, including cellular slime molds (Peng et al., 2012; Amses et al., 2022). In plants, PEPC plays important roles in photosynthesis and anaplerosis for TCA cycle (Podestá and Plaxton, 1994a; 1994b; Chollet et al., 1996). These metabolic functions are pivotal for plant growth and development (Abergel and Glick, 1988; Shi et al., 2015). In *Escherichia coli* and the protozoan *Plasmodium falciparum*, the bacterial-type PEPC plays a role in anaplerotic carbon metabolism, which is required for optimal cell growth (Ashworth and Kornberg, 1966; Chao and Liao, 1993; Peng et al., 2004; Storm et al., 2014). In *D. discoideum*, high-throughput phenotyping of restriction enzyme-mediated integration (REMI) mutants (Sawai et al., 2007) isolated a REMI strain (V30188) harboring a plasmid insertion in the PEPC gene that exhibited aberrant cAMP waves and developmental delay. However, nothing is known about the role of PEPC in *D. discoiduem* development.

In this study, to clarify the functions of PEPC in *D. discoideum* cell differentiation, we constructed a knockout of the PEPC gene by homologous recombination and examined its development as a stand-alone and in chimeras with the parental wild-type. We found that the knockout cells were able to form fruiting bodies with immature spores that appeared ‘glassy’. In chimeras with the wild-type strain, knockout cells were strongly biased toward stalk cell differentiation. Furthermore, using reporter genes and HaloTag based pulse chase assays, we clarified the relationship between early cell–cell heterogeneity in PEPC promoter activity and cell type differentiation.

## 2 Materials and methods

### 2.1 Cell strain, growth and development conditions

*Dictyostelium discoideum* wild-type strain Ax2 and PEPC (dictyBase gene ID: DDB_G0287723) knockout cells were cultured axenically in HL5 medium containing 1.5% (w/v) glucose unless otherwise noted either on a dish or in a shaken flask at 22°C. *pepc* null mutants were generated in the Ax2 background by homologous recombination in the PEPC coding region using a knockout vector isolated from a REMI mutant in an earlier study (Sawai et al., 2007). The vector consisted of the plasmid pBSR1 (Shaulsky et al., 1996) backbone, including a blasticidin resistance cassette flanked by 369 bp and 156bp fragments of the PEPC coding region. Of the 3 knockout strains obtained and verified by Southern blotting, a single clone was selected for analysis. The strain was grown in HL5 medium supplemented with 10 μg/mL blasticidin S. Strains transformed with plasmids having neomycin and/or hygromycin resistance cassette were selected in growth medium supplemented with 10 μg/mL G418 and/or 60 μg/mL Hygromycin. To study the growth rate, cells were inoculated into fresh HL5 medium at a density of 1 × 10^5^ cells/mL in a 50 mL tube and counted every 24 h for 5 days using a hemocytometer. To observe development, the cells were collected, washed with phosphate buffer (PB: 12 mM KH_2_PO_4_, 8 mM Na_2_HPO_4_, pH 6.5), and adjusted to a density of 2.0 × 10^7^ cells/mL. Five microliters of the cell suspension were plated per spot on a water agar plate containing 2% purified agar. Cells were allowed to attach to the agar before removing residual buffer and incubated at 22°C. To count detergent-resistant spores, five pieces of agar were cut and transferred to 5 mL of spore buffer (0.1% TritonX-100, 20 mM EDTA in PB) (Madgwick et al., 2018). After mixing, the number of spores was counted using a hemocytometer.

### 2.2 Multiple sequence alignment and construction of a phylogenetic tree

The amino acid sequences of PEPCs obtained from GenBank and UniProtKB were aligned using ClastalW. ESPript 3.0 was employed for graphical representation of the aligned sequences. A phylogenetic tree was constructed with full-length amino acid sequences by the MEGA X program (Kumar et al., 2018; Stecher et al., 2020) using the neighbor-joining method (Saitou and Nei, 1987). Accession numbers for the obtained amino acid sequences are summarized in Supplementary Table S1.

### 2.3 Plasmid construction

A PEPC promoter fragment (−594 to +12) was amplified by PCR using genomic DNA as the template. The amplified DNA was inserted into the respective pDM vectors upstream of the sequence encoding either GFP, Achilles (Yoshioka-Kobayashi et al., 2020), labile-Achilles (Hashimura et al., 2024), or HaloTag (Arai et al., 2010; Matsuoka et al., 2012). To construct the PEPC expression plasmid, the *act15* promoter in pDM1209 was removed from the XhoI/BglII sites and swapped with a PEPC promoter fragment (−594 to −1). The full-length coding region of the PEPC gene of wild-type, including a single intron, was PCR-amplified from genomic DNA. For the mutant alleles, catalytically dead mutations were introduced using overlap extension PCR. These were inserted into the plasmid at the BglII/SpeI site immediately after the PEPC promoter sequence. To study cell type differentiation, we constructed plasmids that drive the expression of the fluorescent proteins GFP, RFP, Achilles, or mCherry under prestalk-specific *ecmA* or prespore-specific *pspA* promoters. Ax2 and *pepc-* cells were transformed with purified plasmid DNA and selected with antibiotics described above. Stable clones were grown and maintained in the HL5 medium supplemented with antibiotics. For imaging both PEPC protein and promoter activity, we generated a knock-in that harbors a C-terminus insertion of Achilles in the PEPC allele with the CRISPR/Cas9 vector pTM1285 following an earlier study (Yamashita et al., 2021) and chose a single clone for analysis. Primer sequences are summarized in Supplementary Table S2.

### 2.4 Knockout mutant verification by reverse transcriptase PCR and Southern blot analysis

Wild-type Ax2 cells and *pepc* knockout isolates were axenically cultured, collected, and washed twice with PB. Cells were lysed and total RNA was extracted using an automated instrument (Maxwell 16 Instrument AS2000, Promega) in commercially available reaction wells (Maxwell 16 LEV simplyRNA Cells Kit, AS1270, Promega). cDNA was synthesized using reverse transcriptase (SuperScript III First-Strand, 18,080–051, Invitrogen), and PCR was performed using PEPC gene-specific primers (Supplementary Table S2); ribosomal *rnlA* (Ig7) primers were used as controls. Specific PCR products and their sizes were confirmed by gel electrophoresis.

For Southern blot analysis, the genomic DNA of Ax2 and *pepc* null clones was extracted using proteinase K following a modified quick protocol (Charette and Cosson, 2004). Namely, vegetative cells grown in shaken culture were washed twice with phosphate buffer and resuspended in lysis buffer (1M Tris-HCl (pH8.3), 2M KCl, 1M MgCl_2_, 10% Nonidet P-40, 10% Tween 20) containing 0.1 µL proteinase K (QIAGEN, 19,131) per 25 µL lysis buffer. The samples were placed on a thermal cycler at 56°C for 20 min and then at 95°C for 10 min to inactivate proteinase K. After ethanol precipitation, purified genomic DNA was digested with EcoRI for agarose electrophoresis. For Southern blot analysis, the size separated DNA was transferred to a nylon membrane (Amersham Hybond™-N+, RPN1210B, GE HealthCare). The expected fragment sizes, 527 bp in Ax2 and 4,677 bp in *pepc* null mutants, including pBSR1, were confirmed with a PEPC*-*specific probe–PCR amplified PEPC DNA fragment (+600 to +966) labeled with horseradish peroxidase (HRP) using a commercially available kit (Amersham ECL TM Direct Nucleic Acid Labeling and Detection Systems RPN3000OL1, 17,151,028, GE HealthCare) according to the manufacturer’s protocol. Chemiluminescence from the hybridized probe was detected using a digital imager (ImageQuant LAS 4000; GE HealthCare).

### 2.5 Measurement of intracellular oxaloacetate and ATP levels

Intracellular oxaloacetate concentrations were measured using a commercially available assay kit (Amplite Fluorimetric Oxaloacetate Assay Kit Red Fluorescence, 13,841; AAT Bioquest) according to the manufacturer’s protocol. Growing cells were collected at the density of 1.5–1.8 × 10^7^ cells/mL and lysed with a sonicator (TOMY UD-201 ultrasonic disruptor). To remove undisrupted cells and debris, the suspension was passed through a 10 kDa filter (Amicon Ultra-4, UFC801008, Merck Millipore). Additionally, 50 µL of the lysate was used for the oxaloacetate assay. The fluorescence intensity was measured for 1 s using a plate reader (ARVO 1420, Perkin Elmer) with a 531/25 nm excitation filter (531DF25) and 575 nm a long path filter (BA575IF, Olympus). A calibration curve was obtained by measuring a dilution series of the oxaloacetate standard solutions. To measure cellular ATP levels, exponentially growing cells were collected and suspended in HL5 medium at a density of 2 × 10^5^ cells/mL. Further, 50 µL of cell suspension were mixed with the same volume of CellTiter-Glo2.0 assay solution (G9242, Promega) and dispensed into 96-well plate. The specimens were mixed vigorously by mild shaking for 5 min at room temperature and left on a bench for 30 min to allow the chemical reactions to proceed. The luminescence was measured for 1 s using a luminometer (GloMax, Promega). A calibration curve was obtained by measuring a dilution series of ATP solutions with known molarities. Cellular ATP levels were estimated from luminescence with an assumption that the geometry of vegetative cell can be approximated by a 10 µm diameter sphere.

### 2.6 Quantification of the number of prespore and prestalk cells in migrating slug

To count the prestalk and prespore cells, wild-type Ax2 cells and *pepc-* cells were transformed with cell–type reporters: ecmAO:Achilles (prestalk) and pspA:mCherry (prespore) plasmids. Transformants were selected using antibiotics and clones were isolated. Cells developed for 16 h were collected by transferring the migrating slugs to 1 mL of PB containing 20 mM EDTA. The cells were mechanically dissociated by running them back and forth through a 23G needle (NN-2332R, Terumo) 10 times using a syringe. The cell suspension was plated on a cover slip and fluorescence signals were observed under a confocal microscope. Prestalk and prespore cells in the obtained images were counted using ImageJ by manually registering the yellow and red fluorescent cells using the Cell Counter plug-in.

### 2.7 Immunostaining of prespore vacuoles (PSV)

For the fixation of whole mount of slugs, 30–50 µL of PB was dropped on the spot where the cells were developed for 16 h, and the slugs then was floated on the surface of the drop. By placing a coverslip in contact with the surface of PB, the slugs were attached to the coverslip. Subsequently, they were fixed in whole mount with 30, 50, and 100% methanol for 5 min each. After washing by immersing in PBS (137 mM NaCl, 2.68 mM KCl, 10 mM Na_2_HPO_4_·7H_2_O, 2 mM KH_2_PO_4_, pH 7.4), an excess water was removed with paper. To stain individual cells, the mechanically dissociated cells were pelleted by centrifugation and resuspended in 50% methanol. The cells were re-pelleted, resuspended in 100% methanol, and plated onto a glass slide for drying. Both samples, dissociated cells and whole mount slugs, were incubated overnight with the x1,000 diluted anti-PSV monoclonal antibody (a gift from Dr. Yasuo Maeda) at 4°C. After washing with PBS gently, they were incubated overnight with x2000 diluted Alexa488 conjugated anti-mouse IgG antibody at 4°C.

### 2.8 Chimera development

Exponentially growing Ax2 cells carrying pDM1210 act15p:RFP and *pepc-* cells carrying pDM1209 act15p:GFP were washed, mixed at a ratio of 9:1, and developed on water agar plates containing 2% purified agar. As a control, Ax2 cells carrying pDM1209 act15p:GFP were used instead of *pepc-* cells and were mixed with pDM1210 act15p:RFP/Ax2 cells at a ratio of 9:1. Ax2 cells were mixed with the PEPC^OE^ strain pDM1209 act15p:PEPC*-*GFP/pDM358 pspA:mCherry/Ax2 cells and developed together at a 9:1 ratio. Fluorescence intensities were analyzed using Max intensity Z-projected images. pspA:mCherry negative and positive regions were defined based on mCherry signals in that images, and act15p:PEPC-GFP signal within these regions was quantified.

### 2.9 FACS analysis

Flow cytometry and cell sorting were performed using a cell sorter (SH800; Sony). Clonal cells carrying pDM326 PEPCp:Achilles were harvested during the exponential phase (1.5–2.0 × 10^6^ cells/mL) and washed with PB immediately prior to analysis. Achilles fluorescence was detected using a preset EYFP channel. For calibration, Ax2 cells carrying the empty pDM326 plasmid were analyzed as fluorescence-negative populations. The gain level for EYFP signal was set so that the maximum fluorescence intensity (‘EYFP-Height’ value) of the blank cell population is kept below 10^3^. During the sorting step, 5 × 10^4^ cells in the top 5%–7% and low 5%–7% fluorescence intensities were sorted and suspended in the HL5 medium. The cells were cultured for 14 days in 10 mL HL5 medium with 10 μg/mL blasticidin S. The cell density was maintained between 1.0 × 10^5^ to 2.0 × 10^6^ cells/mL by routine dilution with fresh medium. Sorting quality was evaluated by reanalyzing the fraction of sorted cells in each experiment. For all FACS results presented, we confirmed that >99% of the ‘Low’ population when re-analyzed had ‘EYFP-Height’ value below 10^3^ and >93% of the ‘Top’ population had ‘EYFP-Height’ value higher than 10^3^. For various culture conditions, cells carrying pDM326 PEPCp:labile-Achilles were cultured on a Petri dish for 7 days in HL5 medium with glucose, HL5 medium without glucose, or PB with live bacteria. A fraction of the cells was collected for flow cytometric analysis by washing and resuspension in PB at a density of 5 × 10^6^ cells/mL. The remaining cells were washed with PB, resuspended in HL5 medium containing glucose, and incubated for 24 h for further analysis.

### 2.10 Mitochondria staining

Ax2 and *pepc-* cells were harvested during the exponential phase (1.5–2.0 × 10^6^ cells/mL) and adjusted to a density of 1 × 10^7^ cells/mL after washing with PB. The washed cells were stained for 60 min with MitoTracker Red 580 (M22425, Invitrogen) at the final concentration of 10 nM with moderate shaking. An equal volume of DMSO was used instead for the mock-staining control. The cells were washed five times with PB, resuspended in 1 mL of PB, and used for flow cytometric analysis.

### 2.11 HaloTag pulse chase assay

Cells carrying pDM304 PEPCp:Halo or pDM304 act15p:Halo were collected in the exponential phase, washed with PB, and resuspended in HL5 medium at a density of 2 × 10^7^ cells/mL. The washed cells were stained for 30 min with HaloTag TMR ligand (Promega) at the final concentration of 2.5 µM under moderate shaking. The cells were washed twice with PB and resuspended in 500 µL of PB. After 30 min of incubation, cells were resuspended in PB at 2 × 10^7^ cells/mL. Additionally, 5 µL of the cell suspension were plated on a 2% water agar plate. The cells were allowed to develop into slugs and fruiting bodies after incubation for 18 and 24 h, respectively.

### 2.12 Image acquisition

To image developing mounds, slugs, and fruiting bodies, we followed the protocol described previously (Hashimura et al., 2019). A piece of the sample including agar was cut out from the plate and placed upside down on a 35 mm cover-glass bottom dish (12 mm diameter glass, Iwaki) or on a φ25 mm cover glass (25 mm micro cover-glass, Matsunami) mounted on a metal chamber (Attofluor, Invitorogen). In both cases, the center of the cover glass was pre-attached with a 50 µm height ring-shaped spacer (vinyl patch transparent Ta-3N, Kokuyo). The circular space was filled with liquid paraffin (Nacalai Tesque) for refractive index matching.

## 3 Results

### 3.1 Phylogenetic analysis of *Dictyostelium discoideum* PEPC with other PEPCs

**FIGURE 1:**
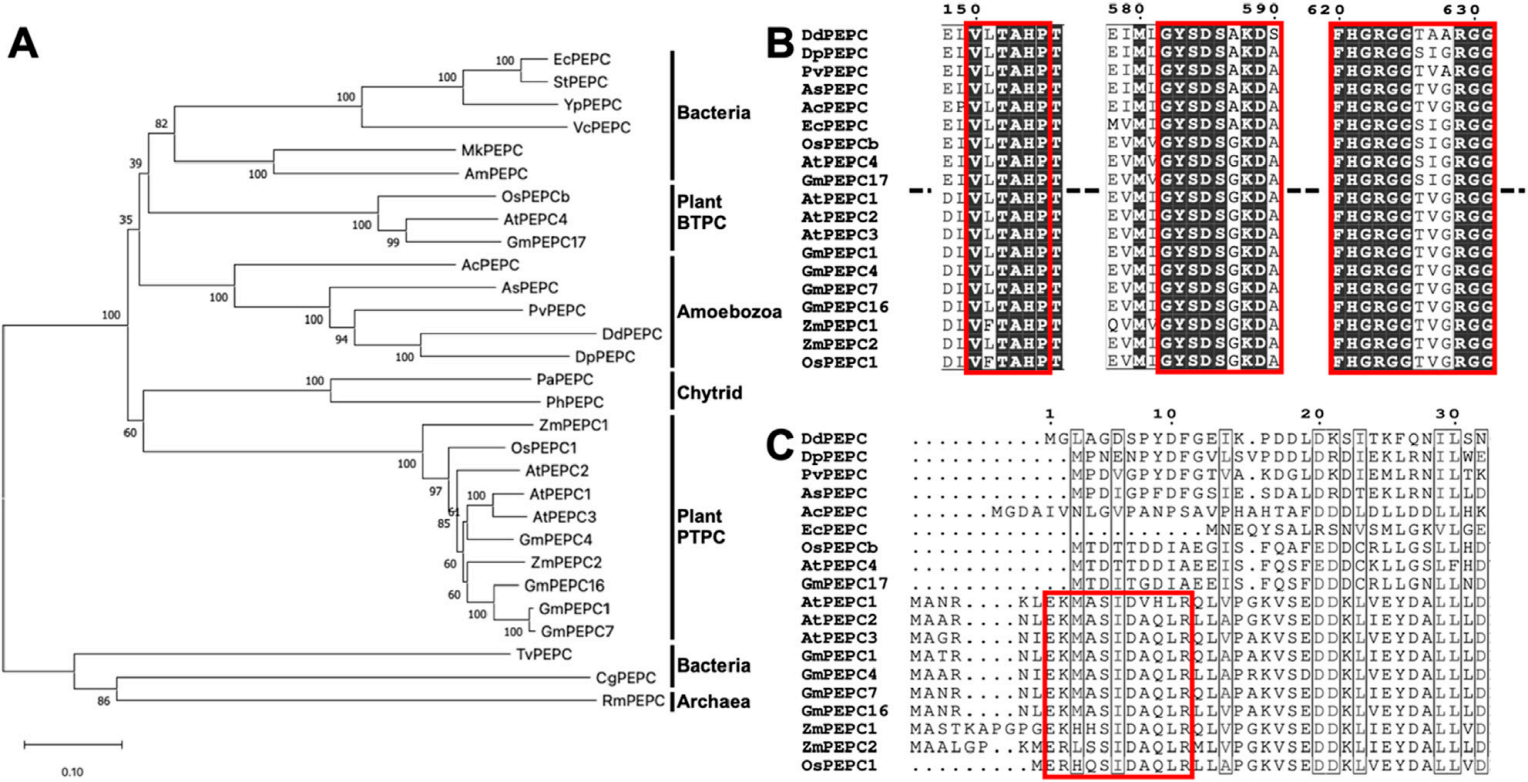
Phylogenetic comparison of DdPEPC with other PEPCs. **(A)** Phylogenic tree constructed with full-length amino acid sequences with the neighbor-joining method (Saitou and Nei, 1987) using the MEGA X program (Kumar S et al., 2018; Stecher G et al., 2020). Numbers indicate the bootstrap values of 1,000 replicates. **(B, C)** Amino acid sequence alignment of selected PEPCs with Clastal W. Output was formatted by ESPript 3. Red box marks the highly conserved domain **(B)** and Plant-type PEPC (PTPC) specific domain **(C)**. Abbreviations: Ec; *Escherichia coli*, St; *Salmonella typhimurium*, Yp; *Yersinia pestis*, Vc; *Vibrio cholerae* serotype, Mk; *Methylonatrum kenyense*, Am; *Alkalispirillum mobile*, Os; *Oryza sativa*, At; *Arabidopsis thaliana*, Gm; *Glycine max*, Ac; *Acanthamoeba castellanii*, As; *Acytostelium subglobosum*, Pv; *Polysphondylium violaceum*, Dd; *Dictyostelium discoideum*, Dp; *Dictyostelium purpureum*, Pa; *Polychytrium aggregatum*, Ph; *Powellomyces hirtus*, Zm; *Zea mays*, Tv; *Thermostichus vulcanus*, Cg; *Corynebacterium glutamicum*, Ro; *Rhodothermus obamensis*.

An earlier phylogenetic analysis (Peng et al., 2012) suggests a complex relationship between eukaryotic and bacterial PEPC. To establish a phylogenetic relationship between *Dictyostelim discoideum* PEPC and other PEPCs from various species, we constructed a phylogenetic tree using the neighbor-joining method with full-length PEPC amino acid sequences from plants, bacteria, chytrids and amoebozoa found in accessible databases (Figure 1A). Amoebozoan PEPCs are found in a large clade of conventional PEPCs, including bacterial- and plant-type PEPCs (Figure 1A). Amoebozoan PEPCs, including *D. discoideum* PEPC (Figure 1; DdPEPC), contain a highly conserved catalytic domain (Figure 1B) and amino acid residues important for catalytic and regulatory functions (Kai et al., 2003) (Supplementary Figure S1). Amoebozoan PEPCs do not have an N-terminal sequence (acid-base-XX-SIDAQLR motif) that characterizes plant PTPC (Figure 1C) (Chollet et al., 1996; Izui et al., 2004), thus mapping close to bacterial-type PEPC and plant BTPC (Ishijima et al., 1986; Sánchez and Cejudo, 2003; Sullivan et al., 2004) (Figure 1A).

### 3.2 Generation and confirmation of PEPC knockout mutant

**FIGURE 2:**
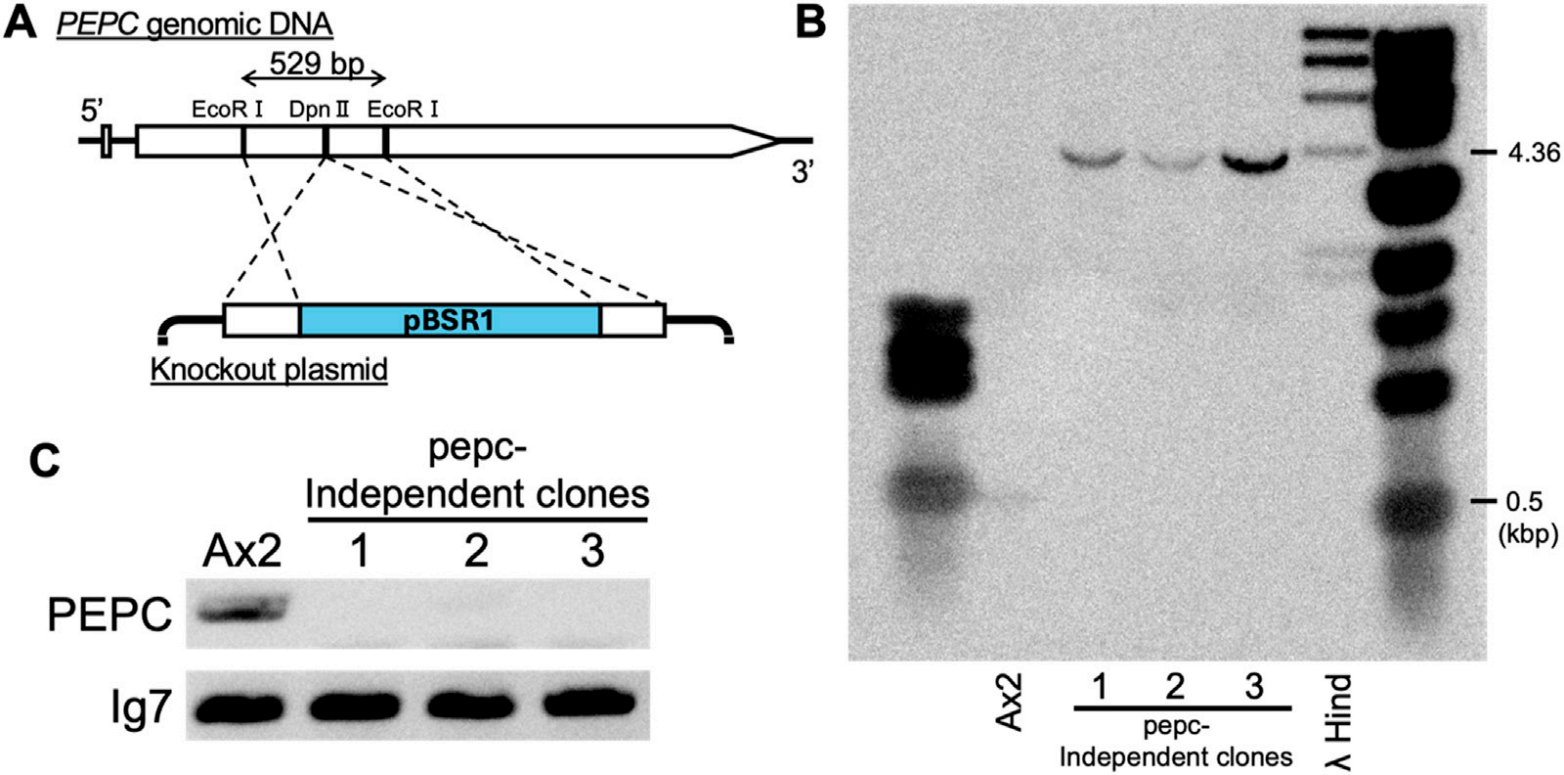
Generation and verification of PEPC null mutant. **(A)** Schematic representation of PEPC disruption by homologous recombination. Knockout vector obtained from a REMI mutant (Sawai et al., 2007) contains the pBSR1 vector backbone and side arms (lower panel) which originate from a 529 bp region flanking a DpnII site (upper panel). **(B)** Verification of homologous recombination by Southern blot analysis. EcoRI digested genomic DNA was probed with PCR amplified EcoRI/DpnII fragment of PEPC. **(C)** Agarose gel electrophoresis of Reverse-Transcriptase PCR product confirming PEPC knockout. Ax2 cell sample shows the expected 1.3 kbp PEPC. Housekeeping gene Ig7 serves as a control.

The developmental phenotype of the PEPC mutant strain of *D*. *discoideum* was first reported in the high-throughput phenotyping of REMI mutants (Sawai et al., 2007); strain V30188. The strain forms aberrant cAMP waves during the cell aggregation stage and exhibits developmental delays. In the present study, to clarify the relationship between PEPC enzymes and cell differentiation, we first recreated the strain with a knockout vector obtained using the plasmid rescue technique (Sawai et al., 2007). The knockout construct consisted of the backbone of the REMI vector pBSR1, which harbors the blasticidin resistance cassette inserted in the coding region flanked by two EcoRI sites in the second exon, whose sequence was identical to the reference genome (Figure 2A). Ax2 cells were electroporated with a vector to obtain PEPC knockout cells by homologous recombination. The transformed cells were grown in axenic medium with blasticidin for selection. After isolating the clonal strains, Southern blot analysis was performed to confirm the insertion of pBSR1 in the target region. Specific signals were detected in both wild-type Ax2 and *pepc-* clone lanes. In *pepc-* lanes, the specific bands were shifted by approximately 4 kbp, close to the expected 4.36 kbp difference (Figure 2B), confirming the insertion of pBSR1 by homologous recombination in the target region. Next, we purified total RNA and performed reverse-transcription PCR using primers specific to the first and second exons of DdPEPC. The results showed that the PEPC-specific signal was only detected in the wild-type Ax2 strain, but not in the selected strains (Figure 2C). This verified that PEPC was not expressed in the three clonal isolates, which we deemed PEPC-null. Strain #1 was used for subsequent analyses.

### 3.3 Disruption of pepc caused the developmental delay and the reduction of spore formation

**FIGURE 3:**
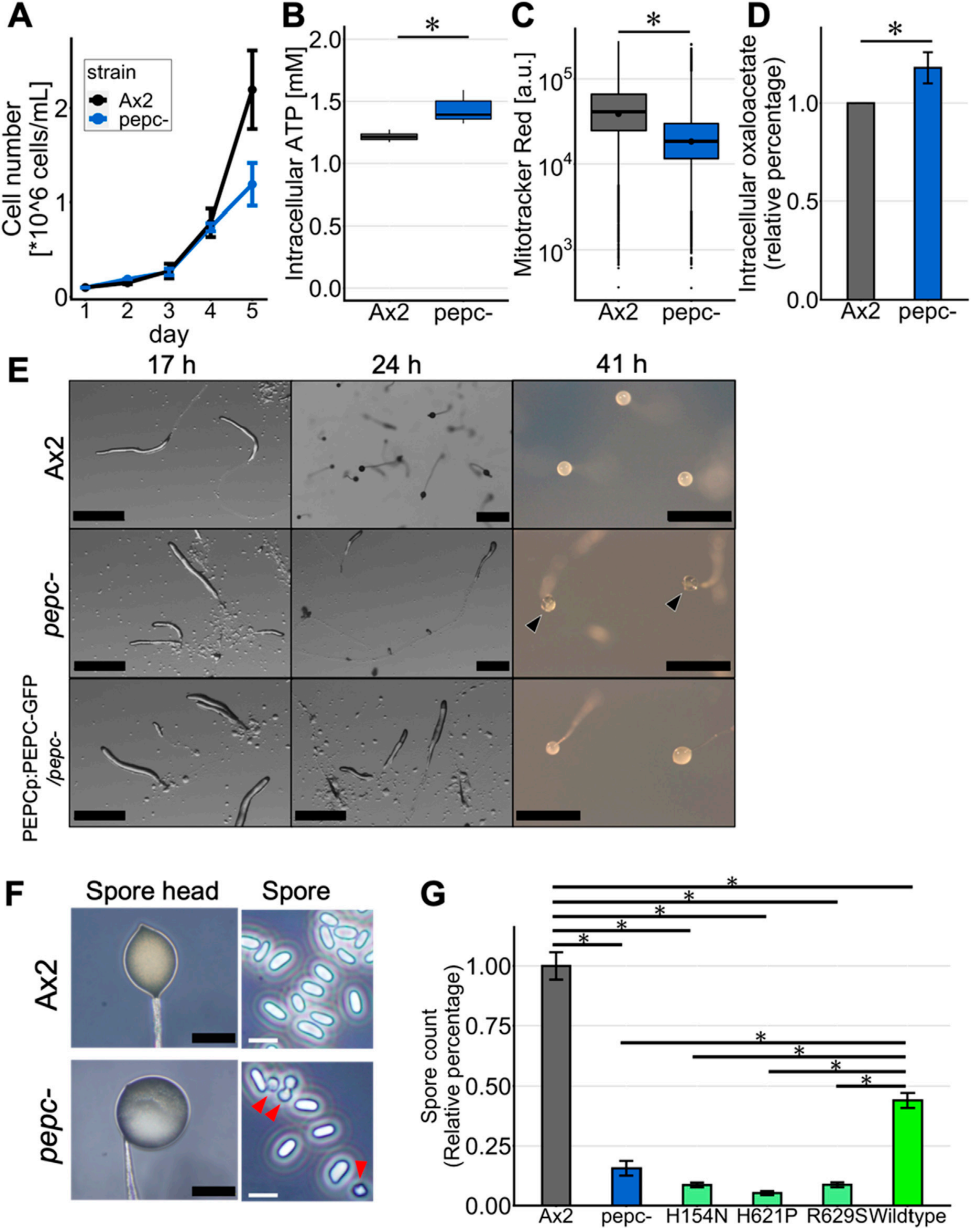
Growth and developmental phenotype of *pepc-* cells. **(A)** Growth of Ax2 and *pepc-* cells cultured in HL5 medium in shaking tube (N = 2). **(B)** Intracellular ATP levels of Ax2 and *pepc-* cells. **(C)** MitoTracker red staining of Ax2 and *pepc-* cells. It stains actively respiring mitochondria (N = 3). **(D)** Intracellular oxaloacetate levels of *pepc-* cells normalized against that of Ax2 (N = 3). **(E)** Multicellular development of Ax2, *pepc-* and PEPC^OE^/*pepc-* cells developed on water agar plate. Black arrow heads indicate “glassy” spore head. Bar: 0.5 mm. (Representative data from N = 3 trials) **(F)** High magnification images of the spore head and isolated spores of Ax2 and *pepc-* mutant. Many *pepc-* spores take round shapes (red arrow heads). Black Bar: 100 μm, White bar: 10 µm. (Representative data from N = 3 trials) **(G)** Average number of detergent resistant spores for *pepc-*, PEPC_H154N_^OE^/*pepc-* (‘H154N’), PEPC_H621P_^OE^/*pepc-* (‘H621P’), PEPC_R629S_^OE^/*pepc-* (‘R629S’) and PEPC^OE^/*pepc-* (‘Wildtype’) normalized against that of Ax2 (N = 3). Data is mean ± s. e. *t*-test; *: p < 0.05.

Vegetative cells grew exponentially in the growth medium under shaking conditions at indistinguishable rates for Ax2 and *pepc-* in the first 4 days. On day 5, *pepc-* showed a significant reduction in the growth rate, suggesting that *pepc-* can grow at the same rate as the parental Ax2 strain; however, its growth was hindered early at relatively low cell densities (Figure 3A). Investigating changes in the metabolic state of *pepc-* cells, the intracellular ATP concentration in *pepc-* cells measured by the luciferase assay was 1.17 times higher (Figure 3B), but the fluorescence intensity of MitoTracker Red, which stains actively respiring mitochondria as measured by flow cytometry, was decreased in *pepc-* cells (Figure 3C). We also measured oxaloacetate levels, which are metabolites generated from phosphoenolpyruvate by PEPC. Unexpectedly, intracellular oxaloacetate levels in *pepc-* cell were 1.23 times higher than those in Ax2 cells (Figure 3D).

Next, we analyzed the development of *pepc-* on water agar plates. The *pepc-* cells showed a mild delay of 2–4 h in the aggregation stage and formed slugs that appeared indistinguishable from Ax2 cells (Figure 3E, 17 h upper panel), except that there were some stalk-like ligaments at the base of the first finger (Figure 3E; 17 h middle panel). The slug persisted longer thus the *pepc-* is a “slugger”, and there was no formation of fruiting bodies after 24 h into starvation (Figure 3E; 24h). Compared to Ax2 wild-type cells that formed mature fruiting bodies after 24 h and remained so for at least 17 h (Figure 3E upper panels), very few fruiting bodies were formed in *pepc-* cells (Figure 3E; 41 h middle panel). Fruiting bodies of *pepc-* had a clear spore head, often described in the literatures as “glassy” (Figure 3E, black arrowhead). When we expressed PEPC fused to Green Fluorescent Protein (GFP) under its native promoter in the *pepc-* background, PEPC-GFP expressing cells still showed developmental delay (Figure 3E; 24 h bottom panel), but the final fruiting bodies had a non-translucent spore head (Figure 3E; 41 h bottom panel). Some spores of *pepc-* had a round shape (Figure 3F, red arrowhead), which was distinct from the mature cylindrical spores. This partially aberrant spore shape is reminiscent of spores in the null-mutant of histidine kinase *dhkB* which also exhibits “glassy” phenotype (Zinda and Singleton, 1998). Other known mutants with “glassy” phenotype such as *stlA-* (Narita et al., 2011) and *hd-* (Myre et al., 2011) exhibit more severe non-encapsulated morphology suggesting that inefficiency of spore maturation in *pepc-* is relatively moderate. To assay spore maturity, we collected spore heads from Ax2 and *pepc-* fruiting bodies and counted their detergent resistance (see Materials and Methods). The number of detergent-resistant spores in *pepc-* mutant was less than 20% of that in the wild-type (Figure 3G). In *pepc-* cells expressing point-mutated PEPC at H154, H621, and R629, homologous residues, which are highly conserved and known to be important sites for enzyme activity (Terada and Izui, 1991; Terada et al., 1991; Yano et al., 1995), the *pepc-* phenotype was not rescued. *pepc-* cells expressing the wild-type allele had approximately three times more detergent-resistant spores than *pepc-* cells, indicating that the developmental phenotype was due to the loss of PEPC catalytic activity. PEPC was not confirmed by Western blot, thus the difference we observed can be due to altered expression level possibly due to use of extrachromosomal vectors. The fact that the WT allele rescued spore count only partially (Figure 3G) points to a possibility of an epigenetic effect.

### 3.4 PEPC gene expression in growth and multicellular stage

**FIGURE 4:**
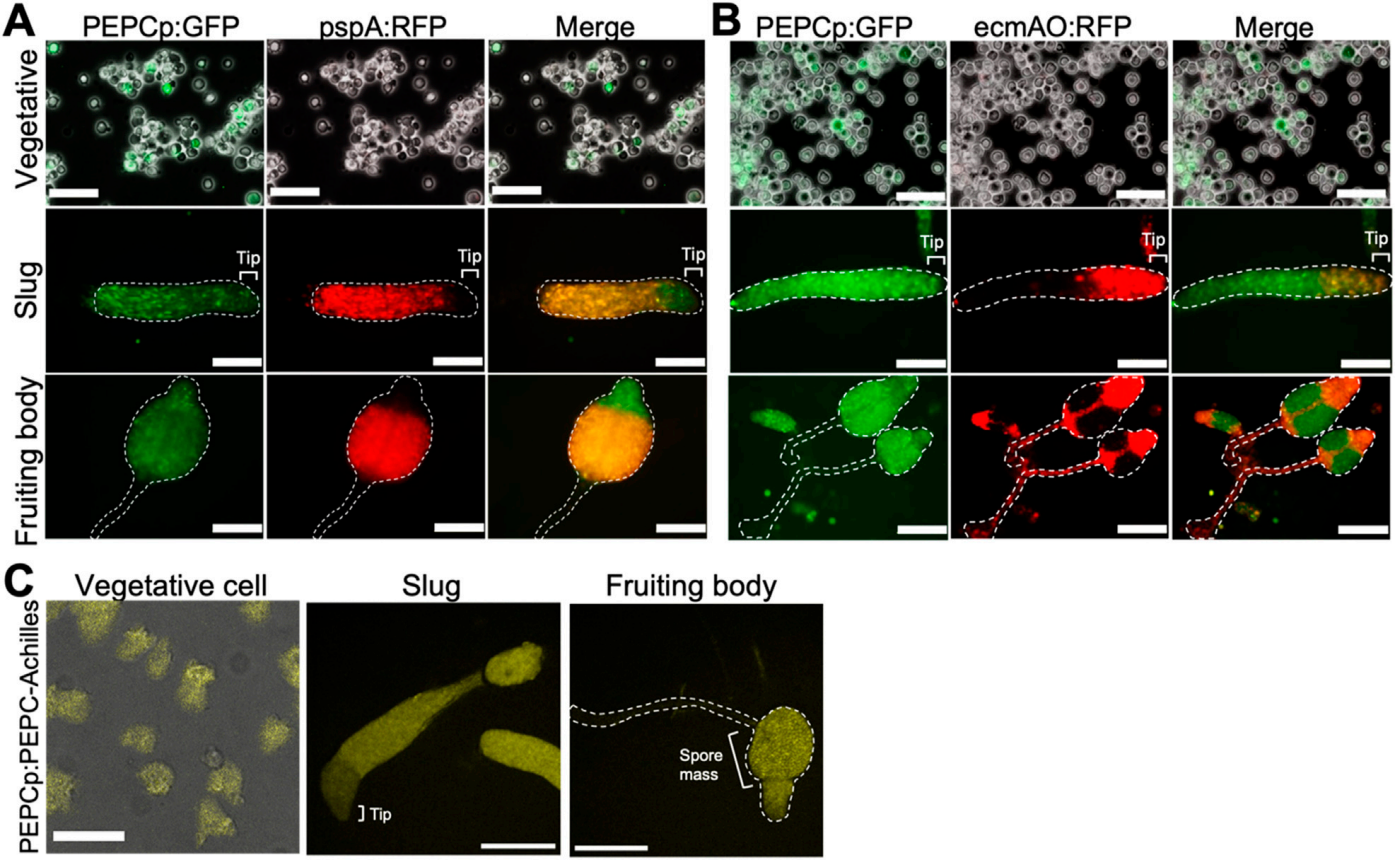
PEPC expression in vegetative and multicellular stages **(A, B)** GFP expression under PEPC promoter (left panels). RFP expression under prespore-specific pspA **(A)** and prestalk-specific ecmAO **(B)** promoter. Vegetative stage (upper panels), slug (middle panels) and fruiting body (lower panels). Bar: 50 µm (Vegetative), 100 µm (Slug and Fruiting body **(A)**), 200 µm (Fruiting body **(B)**) (Representative snapshots from N = 5 trials). **(C)** Knock-in cells expressing PEPC-Achilles from the PEPC allele. Bar: 50 µm (Vegetative), 200 µm (Slug and Fruiting body) (N = 3). Merged images of transmitted light and fluorescence are shown for vegetative data **(A–C)**.

To monitor PEPC gene expression during multicellular development, we amplified its promoter region, including the first 12 bp of the coding sequence (−594 to +12), and used it to construct a GFP expression vector. In addition, the construct contained sequences that drive RFP expression under the prestalk- and prespore- specific promoters ecmA and pspA. In vegetative Ax2 cells harboring the dual expression vectors, we observed heterogeneous GFP fluorescence (Figures 4A,B, PEPCp:GFP upper). For RFP, no fluorescence was detected for the ecmA and pspA promoters, indicating that there was no precocious cell differentiation (Figure 4A pspA:RFP upper, 4B ecmAO:RFP upper). At the slug stage, GFP-expressing PEPC-positive cells were scattered throughout the migrating slug (Figures 4A, B Slug, PEPCp:GFP). When comparing the prestalk (Figure 4A, Slug ecmAO:RFP) and prespore regions (Figure 4A, Slug pspA:RFP), the GFP fluorescence intensity was somewhat higher in the prespore region. GFP-positive cells were mostly found in the spore mass and upper cup of the fruiting body. In contrast, they were almost absent from the stalk (Figure 4B; fruiting bodies PEPCp:GFP and ecmAO:RFP). To monitor PEPC expression more precisely, we knocked in the Achilles coding sequence at the C-terminus of the PEPC locus using the CRISPR/Cas9 system. During the vegetative stage, Achilles fluorescence was extremely weak and barely detectable (Figure 4C, left), suggesting translational and posttranslational regulation. At the slug stage, strong fluorescent signals were more restricted to the prespore region (Figure 4C, middle), in contrast to promoter activity, which was more broadly distributed (Figures 4A,B; slug). Achilles-positive cells were observed in the spore head of the fruiting body and were completely excluded from the stalk (Figure 4C, right). These results suggest that PEPC expression becomes more confined to the spore cells by both transcriptional and post-transcriptional regulation. Together with the fact that the *pepc-* mutant formed aberrant spores (Figure 3F), these results suggest PEPC’s role in spore differentiation.

### 3.5 Cell differentiation in PEPC null mutant

**FIGURE 5:**
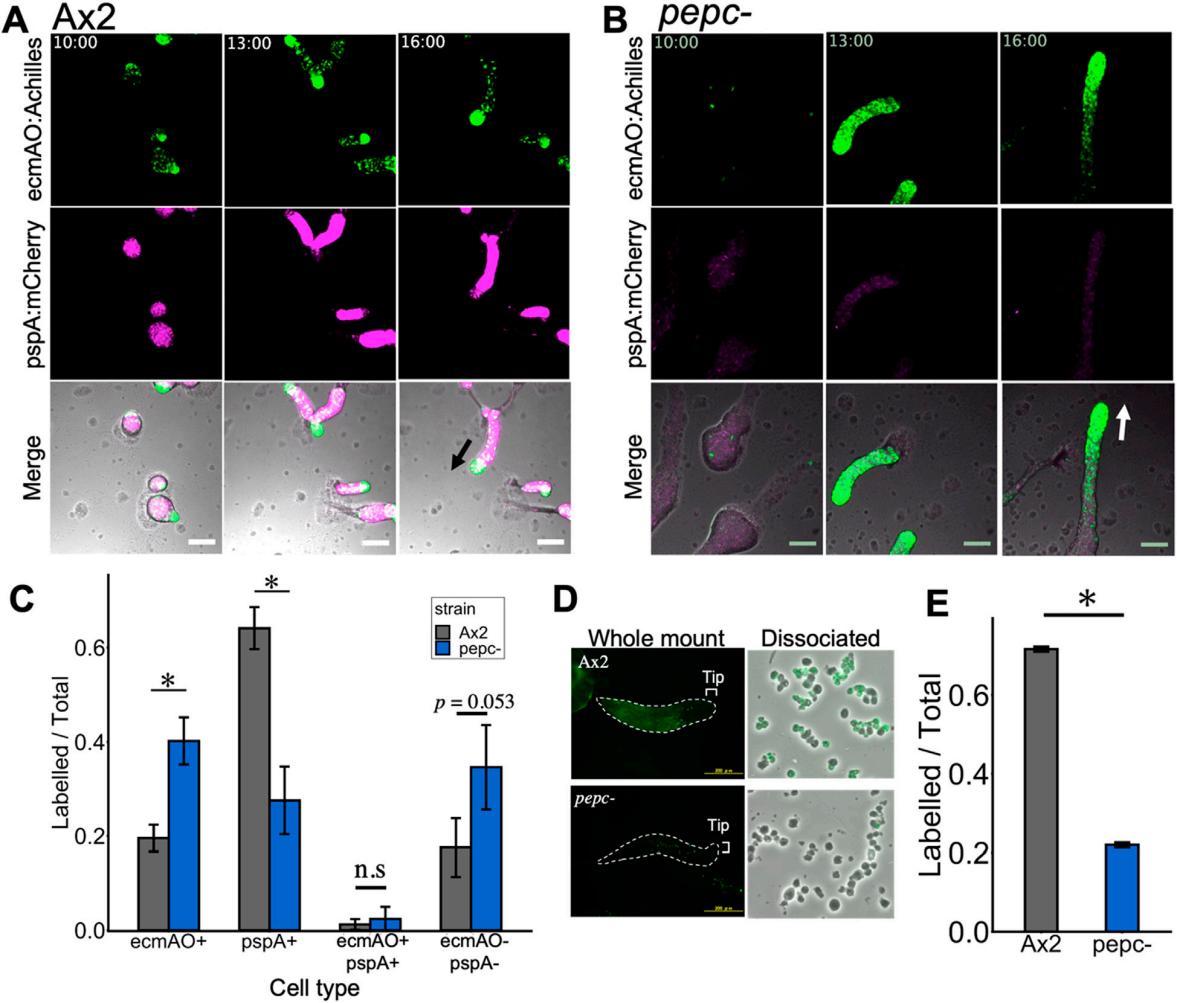
Cell-type differentiation in Ax2 and *pepc-* mutant from mound to early slug stage. **(A, B)** Snapshots from time-lapse imaging of Ax2 **(A)** and *pepc-* **(B)** carrying cell-type specific reporters (green: ecmAO:Achilles, magenta: pspA:mCherry). Merged panels also contain transmitted light images in grayscale (Representative data from N = 4 trials). Black and white arrows indicate direction of slug migration. Numbers in the upper panels indicate time after nutrient removal [hours:min]. Bar: 100 µm. **(C)** Cell-type ratio of cells dissociated from slugs. Cells were counted under a microscope based on the Achilles and mCherry fluorescence (see Materials and Methods). (N = 3; data is mean ± s. e. *t*-test; *: p < 0.05). **(D)** Immunostaining of whole mount slug (left panels) and disaggregated cells (right panels) with a monoclonal anti-PSV (prespore vacuoles) labelled with Alexa488 (green). Upper panels (Ax2) and Lower panels (*pepc-*). Right panels are merged images of Alexa488 fluorescence and transmitted light in grayscale. (Representative data from N = 3 trials). **(E)** Ratio of dissociated cells with positive stain (N = 2). Data is mean ± s. e. *t*-test; *: p < 0.05.

We next examined whether cell differentiation was affected prior to the fruiting body stage. To evaluate cell differentiation, we first employed *pepc-* and Ax2 cells harboring 2 cell type-specific markers, prestalk-specific ecmAO:Achilles and prespore-specific pspA:mCherry. In the wild type, prespore and prestalk cells appeared 8–9 h after starvation (Supplementary Movie S1). Prestalk cells were sorted from prespore cells at 10 h to form the tip region (Figure 5A; 10 h). The mound was elongated to form a migrating slug, where the anterior 1/4 consisted of prestalk cells and the posterior 3/4 consisted of prespore cells. In contrast, *pepc-* cells formed slugs approximately 2 h later than Ax2 cells (Supplementary Movie S2), and remarkably, prestalk cells were found in a graded manner along the anterior-posterior axis without forming a clear boundary between the prespore region. The mCherry-positive cells were scattered throughout the slug, and their fluorescence intensity was weak (Figure 5B). To quantify the promoter activity, cells dissociated from the slug were imaged and counted for Achilles and mCherry fluorescence. We found that the fraction of prespore cells in *pepc-* was reduced by more than 30% compared with that in Ax2 (Figure 5C; pspA+). This was rescued in cells expressing the PEPC protein under the native promoter rescue strain (Supplementary Figure S2). In contrast, prestalk cells increased two-fold in *pepc-* compared to Ax2 (Figure 5C; ecmAO+). Moreover, there was an increase in the number of double-negative cells among *pepc-* cells (Figure 5C; double-). Furthermore, when stained for prespore vacuoles (PSV) using a monoclonal antibody (Tasaka et al., 1983), we found a significant reduction in the number of immunostained cells in *pepc-* (Figure 5D). Accordingly, when dissociated cells were counted, the ratio of immuno-stained cells was reduced by 50% in *pepc-* mutant (Figure 5E). These results suggest that deletion of PEPC inhibits prespore differentiation and favors prestalk differentiation.

**FIGURE 6:**
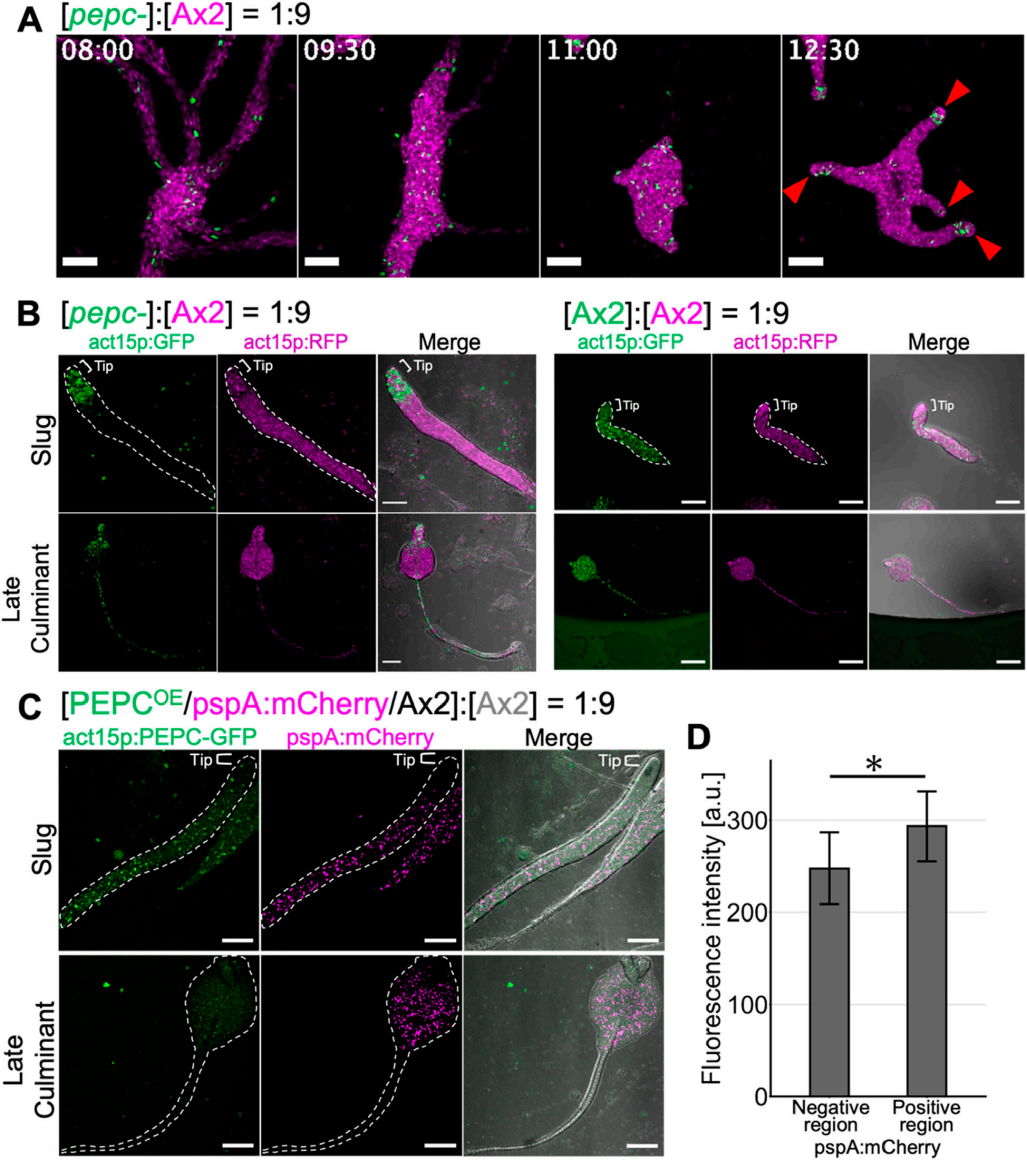
Cell fate bias of *pepc-* and PEPC OE cells in chimera with wildtype Ax2. **(A)** *pepc-* (green: act15p:GFP) and Ax2 (magenta: act15p:mCherry) chimera. Snapshots from time-lapse imaging from streaming to early slug stage. act15p:GFP/*pepc-* (green) and act15p:mCherry/Ax2 (magenta). Red arrow heads indicate the prestalk tip region. Bar: 200 µm. **(B)** *pepc-* (green: act15p:GFP) and Ax2 (magenta: act15p:mCherry) chimera (left panels). Ax2 (green: act15p:GFP) and Ax2 (magenta: act15p:mCherry) chimera (right panels). Slug (upper panels) and culminant stage (lower panels). Bar: 100 µm. **(C)** PEPC^OE^ (green: act15p:PEPC-GFP, magenta pspA:mCherry/Ax2) and Ax2 chimera. Bar: 100 µm. Merged images (right panels) include transmitted light in grayscale **(B, C)**. Representative data from N = 3 trials **(A–C)**. **(D)** Fluorescence intensity of act15p:PEPC-GFP in the pspA:mCherry negative or positive region of slugs developed with PEPC^OE^ and Ax2 cells. N = 9. Data is mean ± s.d. *t*-test; *: p < 0.05.

To address whether the propensity of *pepc-* cells to differentiate into prestalk was a consequence of their reduced prespore differentiation, we performed chimera experiments using *pepc-* and parental Ax2 cells constitutively expressing GFP and mCherry, respectively. The two strains were mixed at a 1:9 ratio (*pepc-* :Ax2) and allowed to develop on a water agar plate. *pepc- *cells were scattered throughout the multicellular body from the streaming stage to the tight mound stage (Figure 6A 8 h–11 h) but began to localize in the anterior prestalk region during slug formation and elongation after cell sorting (Figure 6A 12:30 red arrowhead, Supplementary Movie S3). Accordingly, in later developmental stages, *pepc- cells* cells were found in the anterior region of the slugs, whereas Ax2 cells were found more uniformly (Figure 6B, left panel Slug Ax2). In the late culminant stage, *pepc-* cells were found in the stalk and upper cup regions (Figure 6B, left panel), whereas Ax2 cells were evenly distributed in both the spores and stalks (Figure 6B, left panel Ax2 cell). As a control, Ax2 cells labelled with GFP and mCherry showed no biased distribution in the 1:9 chimera (Figure 6B, right panel). Conversely, when an overexpressor (PEPC^OE^) was mixed with its parental Ax2 cells at a ratio of 1:9 (PEPC^OE^:Ax2), the distribution of PEPC^OE^ cells was slightly biased towards the prespore region marked by pspA:mCherry (Figure 6C Slug), and the fluorescence intensity of PEPC^OE^ cells was 1.2 fold higher in pspA:mCherry positive region (Figure 6D). In the culminant, the GFP signal of PEPC^OE^ cells was weak but still noticeable in the spore head, and continued to express the prespore marker (Figure 6C, Late Culminant). These results suggested that relative difference in PEPC expression had a profound effect on cell fate. Cells that do not express PEPC are strongly biased to differentiate into stalks, whereas those that strongly express PEPC are biased toward spores. This bias appears to be cell-autonomous, as the presence of wild-type cells in the chimera did not rescue the impaired prespore differentiation of *pepc-*.

### 3.6 Pulse-chase analysis of PEPC expressing cells

**FIGURE 7:**
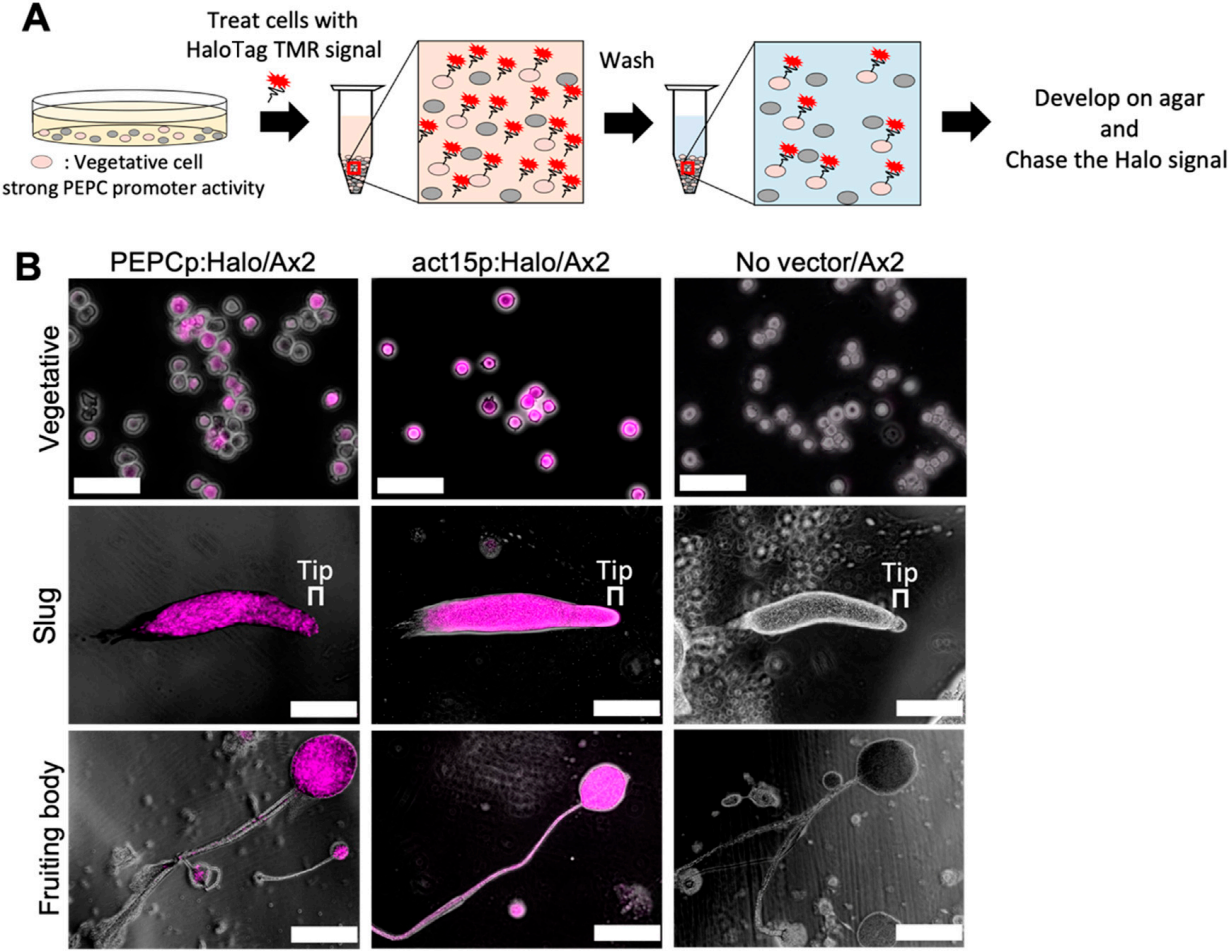
HaloTag pulse chase analysis of cells with early pepc promoter activity. **(A)** Schematic illustration of the pulse chase analysis. HaloTag expressing vegetative cells were labeled with HaloTag TMR ligand and developed on agar (see Materials and Methods). **(B)** Snapshots of HaloTag ligand treated cells. Left panels: PEPCp:Halo/Ax2. Middle panels: act15p:Halo/Ax2. Right panels: negative control (mock transformed Ax2 ‘No vector’). Magenta: HaloTag TMR ligand, Greyscale: transmitted light. Bar: 50 µm (Vegetative), 200 µm (Slug and Fruiting body) (N = 5). Upper panels: cells immediately after staining. Middle panels: slug. Lower panels: fruiting body.

These observations suggest that PEPC expression is necessary for optimal prespore differentiation. Previous studies (Chattwood et al., 2013; Kuwana et al., 2016) have identified two heterogeneously expressed genes associated with cell fate bias. Cells that show high levels of the o-methyltransferase *omt12* during the growth phase have a high tendency to become stalks (Kuwana et al., 2016), and cells expressing low levels of the small GTPase *rasD* are more likely to differentiate into prespore (Chattwood et al., 2013). In this study, we used a haloalkane dehalogenase (HaloTag) that can be covalently labeled with a cell-permeable fluorescent dye (Los et al., 2008). We constructed two strains: one expressing the HaloTag under the PEPC promoter and the other under the *act15* promoter. The growing cells were labeled with a HaloTag-tetramethylrhodamine (TMR) ligand, washed with phosphate buffer, and developed on agar (Figure 7A). Because of the irreversible nature of labeling, cells expressing PEPC at the vegetative stage can be detected and tracked at later multicellular stages (Hashimura et al., 2024). In cells expressing the HaloTag under the control of the PEPC promoter, we found heterogeneous staining in growing cell populations (Figure 7B, PEPCp:Halo/Ax2 Vegetative). The labeled cells were later found in the prespore region of a slug and then almost entirely in the spore head of a fruiting body, with a very minor occurrence in the stalk tube (Figure 7B, PEPCp:Halo/Ax2 Slug and Fruiting body). This heterogeneity was not due to uneven staining, because cells expressing the HaloTag under the constitutive promoter showed uniform fluorescence (Figure 7B, act15p:Halo/Ax2 Vegetative). There was no heterogeneous loss of fluorescent labeling in the slugs and fruiting bodies (Figure 7B, act15p:Halo/Ax2 slugs and fruiting bodies). Wild-type Ax2 cells subjected to the same staining procedure developed normally and showed no detectable fluorescence, indicating that the fluorescent signal originated from the HaloTag ligand (Figure 7B, no vector/Ax2). These results indicate that cells with high PEPC promoter activity in the vegetative stage adopt the spore cell fate.

### 3.7 Glucose and bacteria alter expression of PEPC

**FIGURE 8:**
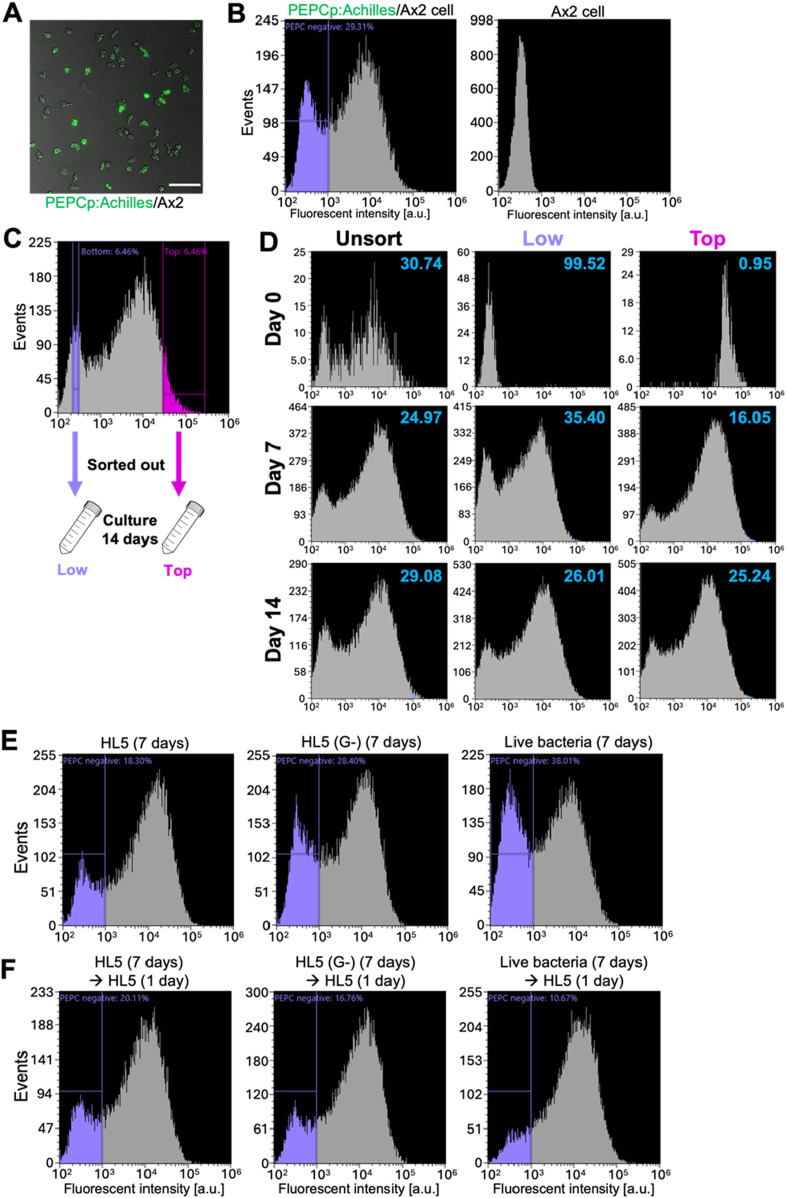
Bimodal PEPC promoter activity in vegetative Ax2 cells **(A)** A snapshot of vegetative Ax2 cells harboring PEPCp:Achilles. Scale bar: 100 µm. **(B)** Fluorescence distribution of PEPCp:Achilles (left panel) and the parental Ax2 cells (right panel). Representative data from N = 3 trials. **(C)** Cells were separated by FACS based on Achilles fluorescence: ‘Top’ (fluorescence positive) and ‘Low’ (fluorescence negative) and grown separately in HL5 growth medium. **(D)** Fluorescence distribution of unsorted (left panels), ‘Low’ population (middle panels) and ‘Top’ populations (right panels) cultured for 0 (upper panels) 7 (middle panels) and 14 days (lower panels). Numbers in the upper right hand side corners indicate the percentage of PEPC negative (Representative data from N = 3 trials). **(E, F)** Fluorescent distribution of PEPCp:labile-Achilles/Ax2 cells cultured in HL5 (left panel), HL5 without glucose (middle panel) and live bacteria (right panel) for 7 days **(E)** and followed by an additional day of culture in fresh HL5 **(F)**. Representative data from N = 3 trials.

To study the nature of PEPC gene expression during the vegetative stage, we performed FACS-based analysis by employing a strain that expresses Achilles under the control of the PEPC promoter (Figure 8A). Cells that expressed almost no Achilles, and thus their fluorescence intensities were only comparable to auto-fluorescence, occupied approximately 30% of exponentially growing populations (Figures 8B, C, fluorescence intensity < 10^3^). Next, we sorted the cells based on Achilles fluorescence and observed changes in their distribution over 14 days (Figure 8C). The cell culture was diluted 10-fold with fresh medium every 2–4 days to maintain a cell density between 1.0 × 10^5^ and 2.0 × 10^6^ cells/mL. In the unsorted control, the fraction of low Achille-expressing cells remained steady at approximately 25%–30% for the duration of our experiments (Figure 8D; Unsorted). Cells sorted for low Achilles expression gradually recovered their expression; the fraction of low-expressing cells decreased from 99.5% to 35.4% by day 7, and then back to 26% by day 14 (Figure 8D; Low). In contrast, in cells sorted for high Achilles expression, Achille-negative cells increased from 1% to approximately 16% by day 7 and eventually occupied approximately 25% of the whole population by day 14 (Figure 8D; Top), suggesting that heterogeneous PEPC expression self-regulates at a ratio of 3:7 (PEPC negative: positive) in the vegetative populations.

These results suggest that extrinsic factors influence the PEPC promoter activity. Early studies (Leach et al., 1973; Thompson and Kay, 2000) have shown that cells grown in a glucose-rich medium have a high tendency to differentiate into spores, similar to PEPC overexpressors. According to RNA-seq data (Gruenheit et al., 2018), expression of PEPC is expected to be reduced in the absence of glucose. To better track changes in Achilles expression after glucose removal, we employed a strain carrying the PEPCp:labile-Achilles expression vector. We found that 18% of the cells were Achilles negative during exponential growth (Figure 8E, left, purple-gated). In the growth medium without glucose, the fraction of PEPC-negative cells increased to 28% by day 7 (Figure 8E, middle). A more pronounced increase in PEPC-negative cells was observed in the live bacterial suspension (Figure 8E, right). After switching the culture conditions back to glucose-containing HL5 medium and incubating for 24 h, we observed that more cells expressed Achilles and the fraction of PEPC-negative cells decreased (Figure 8F). Similar changes were observed when the populations of cells grown on live bacteria suspension were switched to glucose-containing HL5 medium. These results suggest that PEPC promoter activity is environmentally regulated and that glucose-rich conditions, which are known to favor spore differentiation, positively affect PEPC expression.

## 4 Discussion

In the present study, we addressed the role of PEPC in *Dictyostelium*, the first PEPC to be characterized in the Amoebozoa supergroup. Sequence analysis of PEPC in Amoebozoan species showed that the key residues related to its enzymatic activity, including critical sites for tetramerization and cofactor binding, were highly conserved (Supplementary Figure S1). Previous studies (Terada and Izui, 1991; Terada et al., 1991; Yano et al., 1995) have shown that His138, His579, and R587 in *E. coli* PEPC are important for its catalytic activity. The catalytic activity of PEPC appears to be critical for spore differentiation, as the knockout phenotype was not rescued by expressing PEPC harboring a mutation in the homologous residues (Figure 3G). PEPC does not appear to be essential for development because cell aggregation, slug, and fruiting body formation still occur in *pepc-* despite some delay. Rather, the phenotype of *pepc-* marked by excess prestalk, reduced prespore cells, and glassy sporehead (Figure 5; Figures 3E, F), suggests its role in cell differentiation. Notably, *pepc-* cells were also able to form fruiting bodies that appeared normal. It is possible that a fraction of excessive prestalk cells in *pepc-* mutants later differentiated into premature spores or contributed to the basal and lower cups, as has been shown in a deletion mutant of the ABC transporter TagA (Good et al., 2003). Expression of TagA at the onset of starvation is highly correlated with prespore cell fate (Good et al., 2003). Although it is unclear how TagA affects early cell fate, given that it belongs to the ABCB family (Good et al., 2003), it may be involved in mitochondrial ion export, which is crucial for the maintenance of mitochondrial membrane potential. *In vitro* assays have shown that treatment with carbonyl cyanide m-chlorophenyl hydrazone (CCCP) or 2,4-dinitrophenol (DNP), which reduce the proton gradient across the mitochondrial inner membrane, induces prestalk differentiation in the absence of the differentiation-inducing factor DIF-1 (Kubohara et al., 2017). Indeed, *pepc-* cells showed lower mitochondrial membrane potential than wild-type cells (Figure 3C) and were also biased to prestalk cell fate in our chimera assay (Figures 6A,B), suggesting that the cell fate bias exhibited by *pepc-* mutants may be related to altered mitochondrial respiration.

Regarding the developmental delay observed in *pepc-*, the metabolic state surrounding PEPC and its expression may contribute to the timing of the growth/developmental transition. Metabolic flux analyses (Kelly et al., 2021; Mazur et al., 2021) have indicated that mitochondrial respiration decreases during the early stages of *D. discoideum* development, suggesting that starved cells enhance gluconeogenesis rather than promoting the TCA cycle (Mazur et al., 2021). Furthermore, *pck2* (DDB_G0271904), a homologue of phosphoenolpyruvate carboxykinase that catalyzes the reverse reaction of PEPC, was upregulated during the aggregation stage after starvation (Mazur et al., 2021). According to single-cell RNA-seq analysis (Gruenheit et al., 2018), *pck2* and another PCK homologue *pckA* (DDB_G0271678) increased their expression by two-fold when glucose was removed from the medium, regardless of the cell-cycle position. Our present observation that PEPC gene expression is downregulated in the absence of glucose (Figures 8E,F) indicates that there is a coherent regulation of PEPC and PCK expression, which may facilitate a sharp transition in the metabolic state. However, it is somewhat counterintuitive that *pepc-* cells show delayed aggregation and mound formation, as their reduced mitochondrial membrane potential is expected to help the growth/development transition. This may be related to our unexpected observations of elevated oxaloacetate concentration in *pepc-* cells which hint at genetic compensation that alters expression of other oxaloacetate synthesizing enzymes such as malate dehydrogenase and aspartate aminotransferase.

According to RNA-seq analysis (Gruenheit et al., 2018), PEPC is among the 901 genes that are highly expressed in the G2 stage of the cell cycle. According to this study, cells in the G2 stage have a high tendency to differentiate into prespore cells, which agrees with our observation that cells with higher PEPC activity immediately before starvation differentiate into spores (Figure 7B). The vegetative cells with high PEPC promoter activity were larger than those with low activity (Supplementary Figure S3), suggesting that these cells were in the G2 phase. Given that, at the protein level, PEPC-Achilles was barely detectable in the vegetative stage, PEPC expression in G2 cells is likely to be maintained by translational repression or protein degradation unless they are starved.

Since PEPC overexpression (PEPC^OE^/Ax2) resulted in the formation of a complete fruiting body (Supplementary Figure S4), the mechanism that realizes the correct cell type ratio appears to be unaffected by excessive PEPC. As PEPC overexpressor in a 1:9 mixture with wild-type cells showed its tendency to differentiate into spores (Figure 6C), we postulated that cells are preferentially assigned a spore fate based on the relative difference in the metabolic state. Furthermore, PEPC is listed as a spore upregulated gene (Kin et al., 2018) which is consistent with our reporter gene analysis and the stronger appearance of PEPC-Achilles fluorescence in the prespore region (Figure 4). This PEPC expression should further enhance spore differentiation and maturity. It is known that a null mutant of a polyketide synthetase SteelyA (StlA), exhibits ‘glassy’ sori and do not produce 4-methyl-5-pentylbenzene-1,3 diol (MPBD) required for spore maturation (Narita et al., 2011). The presence of immature spores in *pepc-* (Figures 3F,G) suggested that MPBD may be reduced in this mutant. MPBD is likely modified by the O-methyltransferase omt12 (Ghosh et al., 2008) which is a marker for cell fate bias in a subpopulation of stalk cells found at the very tip of a slug (Kuwana et al., 2016). It may be possible that MPBD is consumed to form derivatives that act to increase subset of prestalk cells (Kondo et al., 2019).

The strong correlation observed between PEPC expression and spore differentiation highlights several questions that require future analysis. Our study showed that PEPC promoter activity was suppressed when grown in a liquid growth medium without glucose or live bacteria suspension. What ties cell–cell heterogeneity in glucose metabolism and cell fate bias? In mouse ES cells, glucose metabolic activity controls cell fate during the growth/differentiation transition by promoting epigenetic changes (Moussaieff et al., 2015; TeSlaa et al., 2016). The activities of AMPK and mTORC, which are mutually antagonistic sensors of energy status (i.e., ATP/ADP ratio), influence ES cell fate decisions (Tatapudy et al., 2017; Lu et al., 2021). In *D. discoideum*, cells lacking AMPK exhibited enhanced mTORC1 activity and preferentially differentiated into stalks (Wang Y. et al., 2024). A reduction in mTORC1 activity induces spore cell differentiation, and histone methyltransferase Set1 knockout increases the phosphorylation of the mTORC1 target 4E-BP1(Wang Y. et al., 2024). These observations suggest that cells primed to express PEPC during the vegetative stage have low mTORC1 activity and ATP/ADP ratios. However, this notion does not align well with the fact that an increase in glucose concentration in the growth medium enhances mTORC1 activity and reduces AMPK activity in *D. discoideum* (Jaiswal and Kimmel, 2019). Future studies should address the relationship between cell–cell heterogeneity in PEPC, metabolic flux, and the activities of energy sensors. Clarifying these aspects should help to illuminate both the commonalities and diversity that drive the evolution of cell differentiation and multicellularity (Yamagishi et al., 2016).

## Data Availability

The original contributions presented in the study are included in the article/Supplementary Material, further inquiries can be directed to the corresponding author.
