# Supplementary material for: Cell–cell heterogeneity in phosphoenolpyruvate carboxylase biases early cell fate priming in Dictyostelium discoideum
Source: Front Cell Dev Biol. 2025 Feb 4;12:1526795. doi: 10.3389/fcell.2024.1526795 (PMC11832675; doi:10.3389/fcell.2024.1526795)
Supplement: Supplementary file 3 [file DataSheet1.pdf]

Supplementary Material

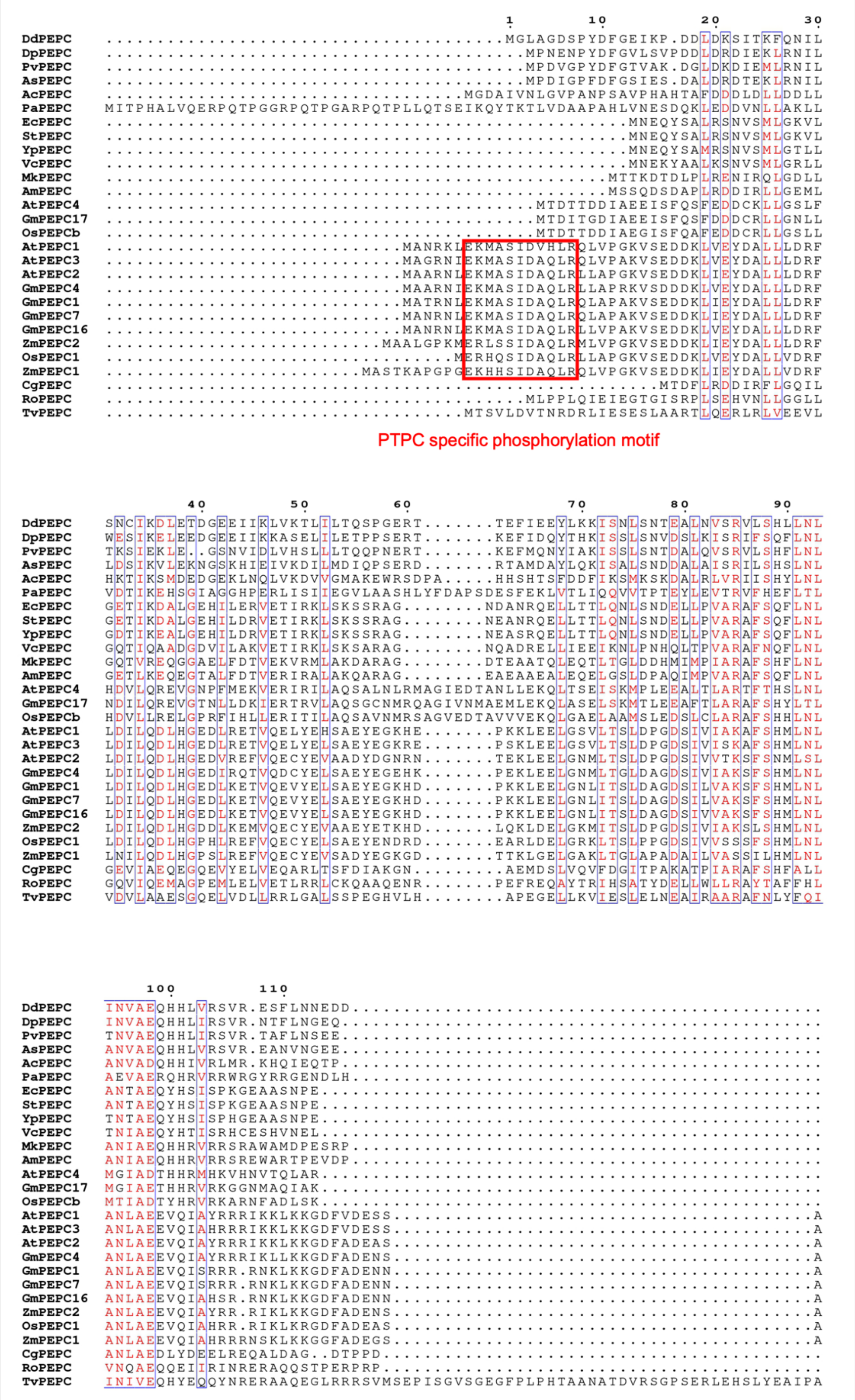

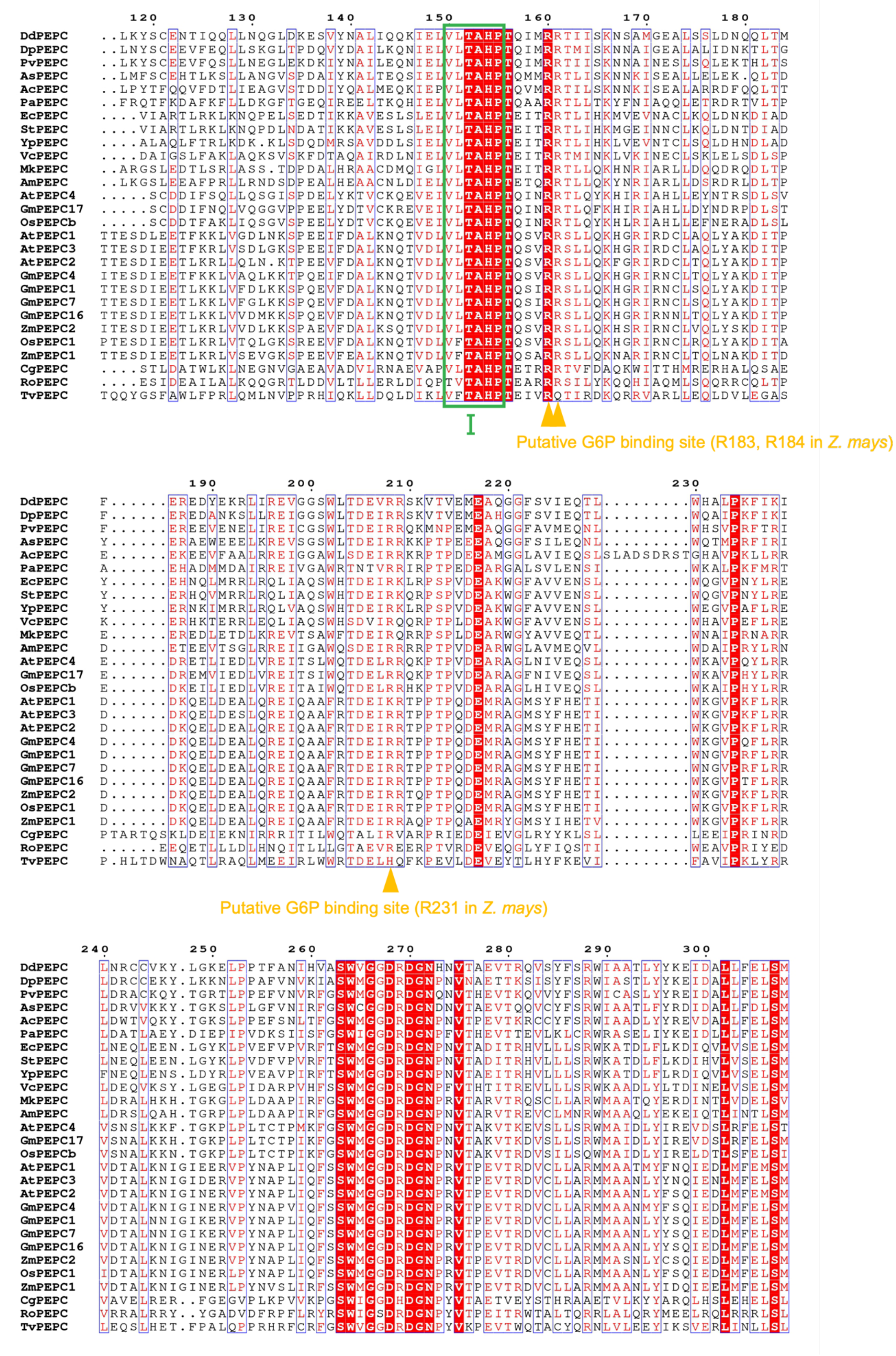
I
Putative G6P binding site (R183, R184 in Z. mays)
Putative G6P binding site (R231 in Z. mays)

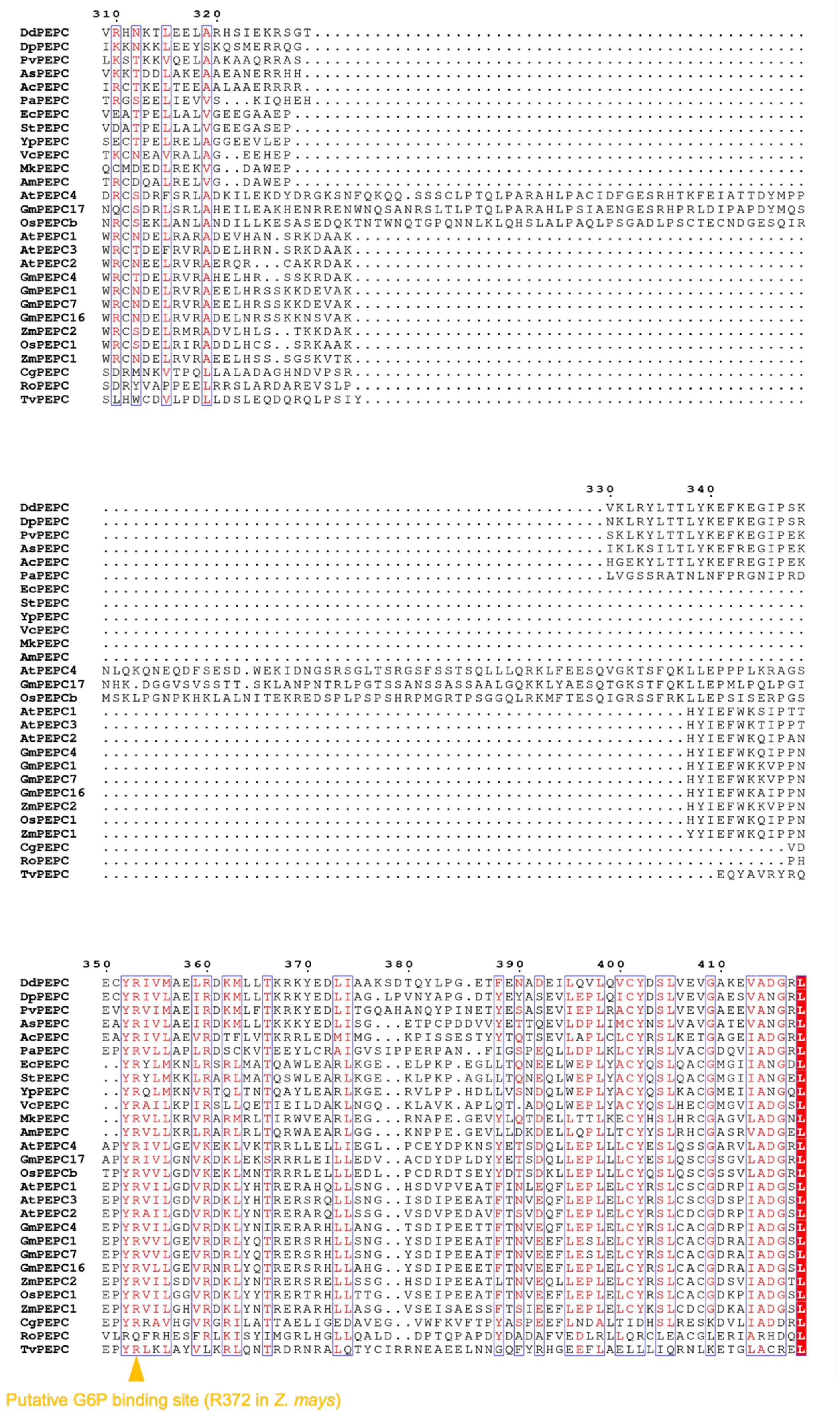
310 320
DdPEPC VRHNKTLEELARHSIEKRSGT
DpPEPC IKKNKKLEEYSKQSMERRQG
PvPEPC LKSTKKVQELAAKAAQRRAS
AsPEPC VKKTDDLAKEAAEANERRHH
AcPEPC IRCTKELTEEAALAAERRRR
PaPEPC TRGSEELIEVVS...KIQHEH
EcPEPC VEATPELLALVGEEGAAEP
StPEPC VDATPELLALVGEEGASEP
YpPEPC SECTPELRELAGGEEVLEP
VcPEPC TKCNEAVRALAG..EEHEP
MkPEPC QCMDEDLREKVG..DAWEP
AmPEPC TRCDQALRELVG..DAWEP
AtPEPC4 DRCSDRFSRLADKILEKDYDRGKSNFQKQQ.SSSCLPTQLPARAHLPACIDFGESRHTKFEIATTDYMPP
GmPEPC17 NQCSDRLSRLAHEILEAKHENRRENWNQSANRSLTLPTQLPARAHLPSIAENGESRHPRLDIPAPDYMQS
OsPEPCb NRCSEKLANLANDILLKESASEDQKTNTWNQTGPQNNLKLQHSLALPAQLPSGADLPSCTECNDGESQIR
AtPEPC1 WRCNDELRARADEVHAN.SRKDAAK
AtPEPC3 WRCTDEFRVRADELHRN.SRKDAAK
AtPEPC2 WRCNEELRVRAERQR...CAKRDAK
GmPEPC4 WRCTDELRVRAHELHR..SSKRDAK
GmPEPC1 WRCNDELRVRAEELHRSSKKDEVAK
GmPEPC7 WRCNDELRVRAEELHRSSKKDEVAK
GmPEPC16 WRCNDELRVRADELNRSSKKNSVAK
ZmPEPC2 WRCSDELRMRADVLHLS..TKKDAK
OsPEPC1 WRCSDELRIRADDLHCS..SRKAAK
ZmPEPC1 WRCNDELRVRAEELHSS.SGSKVTK
CgPEPC SDRMNKVTPQLLALADAGHNDVPSR
RoPEPC SDRYVAPPEELRRSLARDAREVSLP
TvPEPC SLHWCDVLPDLLDSLEQDQRQLPSIY
330 340
DdPEPC VKLRYLTTLYKEFKEGIPSK
DpPEPC NKLRYLTTLYKEFKEGIPSR
PvPEPC SKLKYLTTLYKEFKEGIPEK
AsPEPC IKLKSILTLYKEFREGIPEK
AcPEPC HGEKYLTTLYKEFREGIPEK
PaPEPC LVGSSRATNLNFPRGNIPRD
EcPEPC
StPEPC
YpPEPC
VcPEPC
MkPEPC
AmPEPC
AtPEPC4 NLQKQNEQDFSESD.WEKIDNGSRSGLTSRGSFSSTSQLLLQRKLFEESQVGKTSFQKLLEPPPLKRAGS
GmPEPC17 NHK.DGGVSVSSTT.SKLANPNTRLPGTSSANSSASSAALGQKKLYAESQTGKSTFQKLLEPMLPQLPGI
OsPEPCb MSKLPGNPKHKLALNITEKREDSPLPSPSHRPMGRTPSGGQLRKMFTESQIGRSSFRKLLEPSISERPGS
AtPEPC1 HYIEFWKSIPTT
AtPEPC3 HYIEFWKTIPPT
AtPEPC2 HYIEFWKQIPAN
GmPEPC4 HYIEFWKQIPPN
GmPEPC1 HYIEFWKKVPPN
GmPEPC7 HYIEFWKKVPPN
GmPEPC16 HYIEFWKAIPPN
ZmPEPC2 HYIEFWKKVPPN
OsPEPC1 HYIEFWKQIPPN
ZmPEPC1 YYIEFWKQIPPN
CgPEPC VD
RoPEPC PH
TvPEPC EQYAVRYRQ
350 360 370 380 390 400 410
DdPEPC ECYRIVMAELRDKMLLTKRKYEDLIAAKSDTQYLPG.ETFENADEILQVLQVCYDSLVEVGAKEVADGRL
DpPEPC ECYRIVLAEIRDKMLLTKRKYEDLIAG.LPVNYAPG.DTYEYASEVLEPLQICYDSLVEVGAESVANGRL
PvPEPC EVYRVIMAEIRDKMLFTKRKYEDLITGQAHANQYPINETYESASEVIEPLRACYDSLVEVGAEEVANGRL
AsPEPC EAYRIVLAEIRDKMLLTKKKYEDLISG...ETPCPDDVVYETTQEVLDPLIMCYNSLVAVGATEVANGRL
AcPEPC EAYRIVLAEVRDTFLVTKRRLEDMIMG...KPISSESTYYTQTSEVLAPLCLCYRSLKETGAGEIADGRL
PaPEPC EPYRVLLAPLRDSCKVTEEYLCRAIGVSIPPERPAN..FIGSPEQLLDPLKLCYRSLVACGDQVIADGRL
EcPEPC ..YRYLMKNLRSRLMATQAWLEARLKGE..ELPKP.EGLLTQNEELWEPLYACYQSLQACGMGIIANGDL
StPEPC ..YRYLMKKLRARLMATQSWLEARLKGE..KLPKP.AGLLTQNEQLWEPLYACYQSLQACGMGIIANGEL
YpPEPC ..YRQLMKNVRTQLTNTQAYLEARLKGE..RVLPP.HDLLVSNDQLWEPLYACYQSLKACGMEIIANGQL
VcPEPC ..YRAILKPIRSLLQETIEILDAKLNGQ..KLAVK.APLQT.ADQLWEPLYACYQSLHECGMGVIADGSL
MkPEPC ..YRVLLKRVRARMRLTIRWVEARLEG...RNAPE.GEVYLQTDELLTTLKECYHSLHRCGAGVLADGNL
AmPEPC ..YRVLLKRLRARLRLTQRWAEARLGG...KNPPE.GEVLLDKDELLQPLLTCYYSLRHCGASRVADGEL
AtPEPC4 APYRIVLGEVKEKLVKTRRLLELLIEGL..PCEYDPKNSYETSDQLLEPLLLCYESLQSSGARVLADGRL
GmPEPC17 APYRIVLGNVKDKLEKSRRRLEILLEDV..ACDYDPLDYYETSDQLLEPLLLCYESLQSCGSGVLADGRL
OsPEPCb TPYRVVLGDVKEKLMNTRRRLELLLEDL..PCDRDTSEYYDTSDKLLEPLLLCYQSLQSCGSSVLADGRL
AtPEPC1 EPYRVILGDVRDKLYHTRERAHQLLSNG..HSDVPVEATFINLEQFLEPLELCYRSLCSCGDRPIADGSL
AtPEPC3 EPYRVILGDVRDKLYHTRERSRQLLSNG..ISDIPEEATFTNVEQFLEPLELCYRSLCSCGDSPIADGSL
AtPEPC2 EPYRAILGDVRDKLYNTRERARQLLSSG..VSDVPEDAVFTSVDQFLEPLELCYRSLCDCGDRPIADGSL
GmPEPC4 EPYRVILGDVRDKLYNIRERARHLLANG..TSDIPEETTFTNVEQFLEPLELCYRSLCACGDRPIADGSL
GmPEPC1 EPYRVVLGEVRDRLYQTRERSRHLLSNG..YSDIPEEATFTNVEEFLESLELCYRSLCACGDRAIADGSL
GmPEPC7 EPYRVVLGEVRDRLYQTRERSRHLLSNG..YSDIPEEATFTNVEEFLESLELCYRSLCACGDRAIADGSL
GmPEPC16 EPYRVLLGEVRNRLYQTRERSRHLLAHG..YSDIPEEETFTNVEEFLEPLELCYRSLCACGDRAIADGSL
ZmPEPC2 EPYRVILSDVRDKLYNTRERSRELLSSG..HSDIPEEATLTNVEQLLEPLELCYRSLCACGDSVIADGTL
OsPEPC1 EPYRVILGGVRDKLYYTRERTRHLLTTG..VSEIPEEATFTNVEEFLEPLELCYRSLCACGDKPIADGSL
ZmPEPC1 EPYRVILGHVRDKLYNTRERARHLLASG..VSEISAESSFTSIEEFLEPLELCYKSLCDCGDKAIADGSL
CgPEPC EPYRRAVHGVRGRILATTAELIGEDAVEG..VWFKVFTPYASPEEFLNDALTIDHSLRESKDVLIADDRL
RoPEPC VLRQFRHESFRLKISYIMGRLHGLLQALD..DPTQPAPDYDADAFVEDLRLLQRCLEACGLERIARHDQL
TvPEPC EPYRLKLAYVLKRLQNTRDRNRALQTYCIRRNEAEELNNGQFYRHGEEFLAELLLIQRNLKETGLACREL
Putative G6P binding site (R372 in Z. mays)

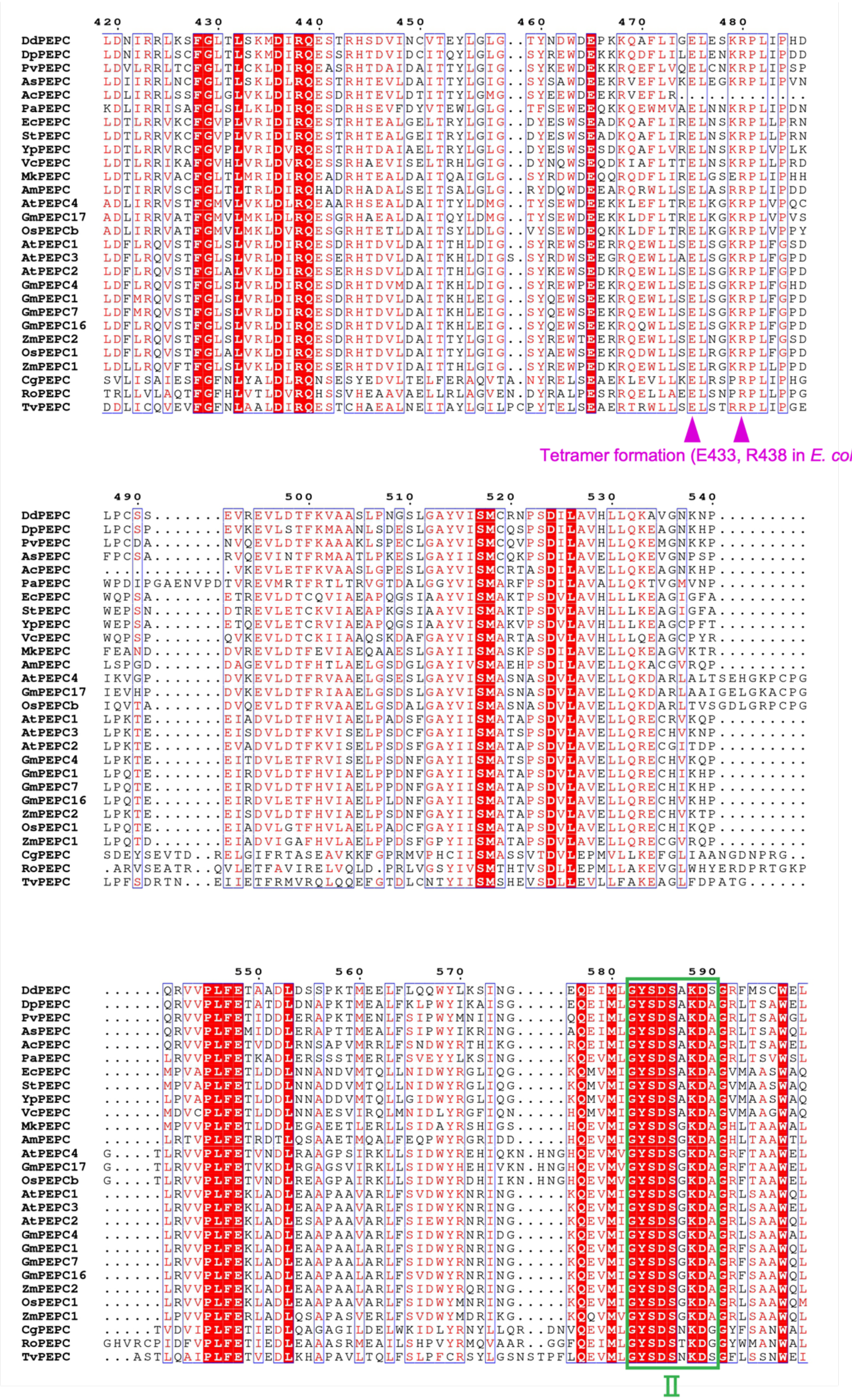
Tetramer formation (E433, R438 in E. coli)
II

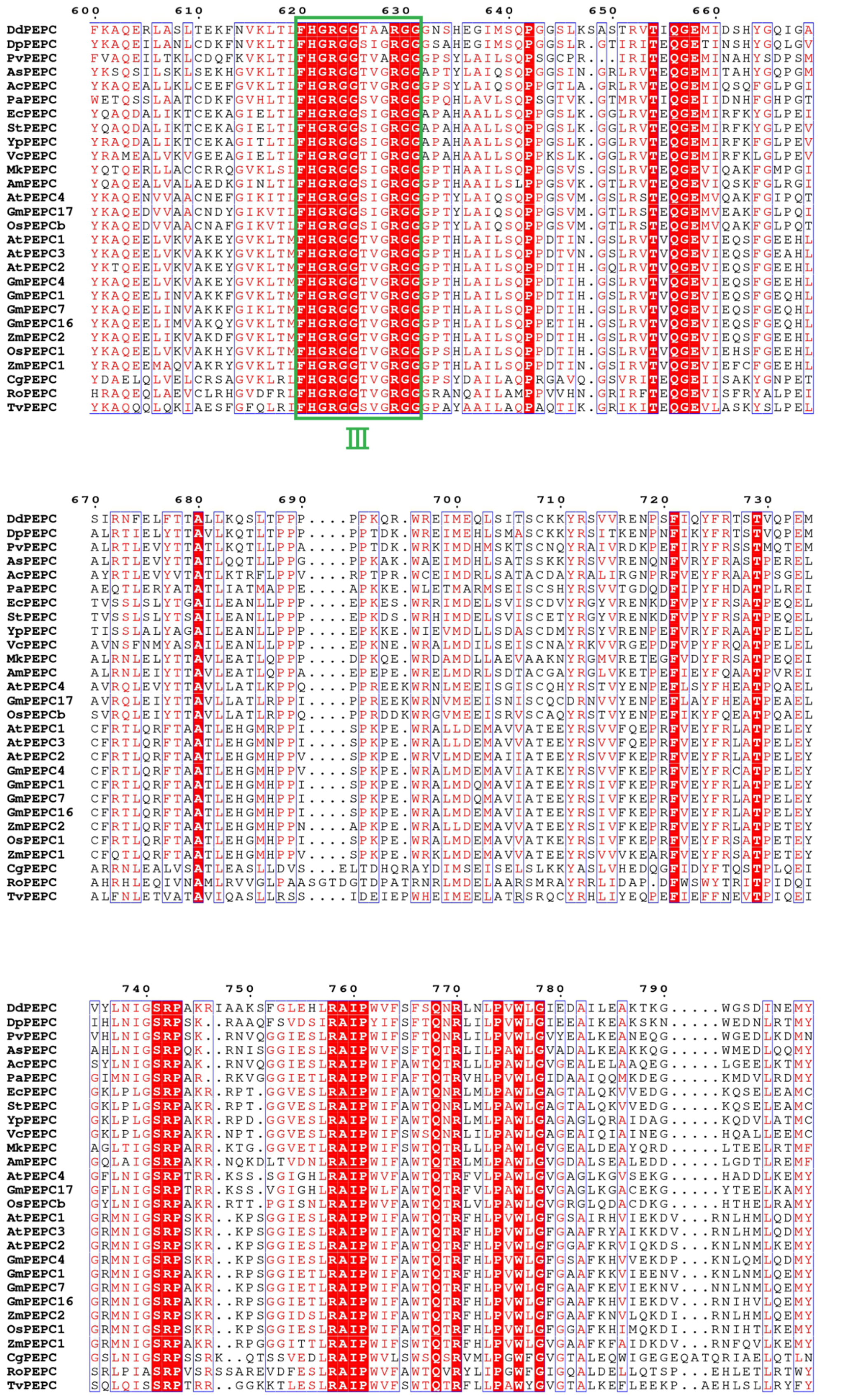
DdPEPC
DpPEPC
PvPEPC
AsPEPC
AcPEPC
PaPEPC
EcPEPC
StPEPC
YpPEPC
VcPEPC
MkPEPC
AmPEPC
AtPEPC4
GmPEPC17
OsPEPCb
AtPEPC1
AtPEPC3
AtPEPC2
GmPEPC4
GmPEPC1
GmPEPC7
GmPEPC16
ZmPEPC2
OsPEPC1
ZmPEPC1
CgPEPC
RoPEPC
TvPEPC
III

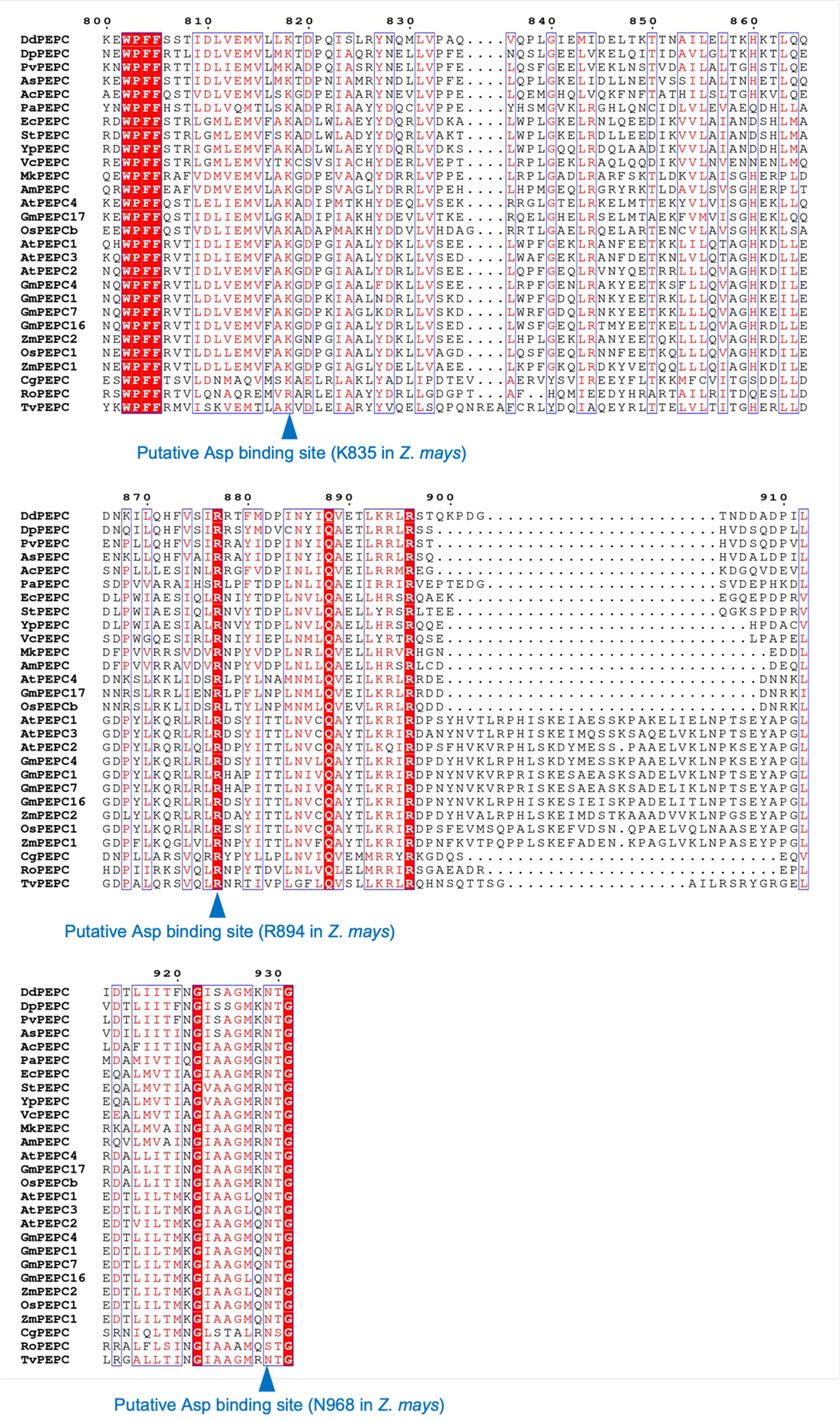
Putative Asp binding site (K835 in Z. mays)
Putative Asp binding site (R894 in Z. mays)
Putative Asp binding site (N968 in Z. mays)

Supplementary Figure 1. Full length amino acid sequence alignment of 28 PEPCs. Full length sequence alignment of 28 PEPCs performed by Clastal W. The output was formatted by ESPript 3. Green boxes I-III denote the conserved subdomains essential for PEPC catalysis (Kai et al., 2003). Ec; *Escherichia coli*, St; *Salmonella typhimu*, Yp; *Yersinia pestis*, Vc; *Vibrio cholerae serotype*, Mk; *Methylonatrum kenyense*, Am; *Alkalispirillum mobile*, Os; *Oryza sativa*, At; *Arabidopsis thaliana*, Gm; *Glycine max*, Ac; *Acanthamoeba castellanii*, As; *Acytstelium subglobosum*, Pv; *Polysphondylium violaceum*, Dd; *Dictyostelium discoideum*, Dp; *Dictyostelium purpureum*, Pa; *Polychytrium aggregatum*, Ph; *Powellomyces hirtus*, Zm; *Zea mays*, Tv; *Thermostichus vulcanus,* Cg; *Corynebacterium glutamicum*, Ro; *Rhodothermus obamensis.*

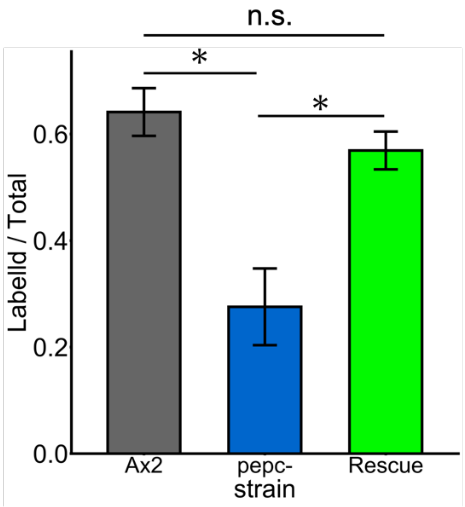

Supplementary Figure 2. Prespore differentiation in Ax2, *pepc*- and *pepc*- resue strain. Ratio of prespore cells evaluated by pspA:mCherry in total cells dissociated from slugs. Prespore cells were counted under a microscope based on the mCherry fluorescence (see Materials and Methods). (N = 3; data is mean ± s.e. t test; *: $p < 0.05$).

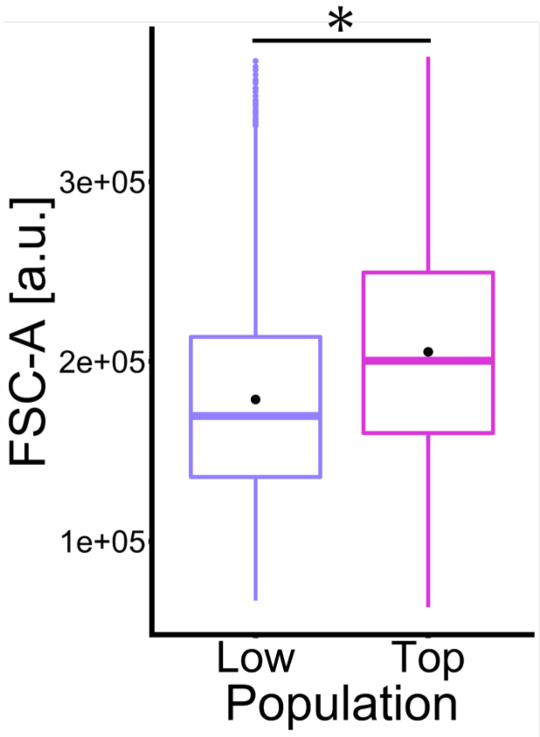

Supplementary Figure 3. Forward scatter of separated cell population. Cells carrying pDM326 PEPCp:Achilles were separated by FACS based on Achilles fluorescence: 'Low' (fluorescence negative) and 'Top' (fluorescence positive) (N = 3 independent experiments; n = 4100 (Low), 2370 (Top); Black dot is mean. t test; *: $p < 0.05$).

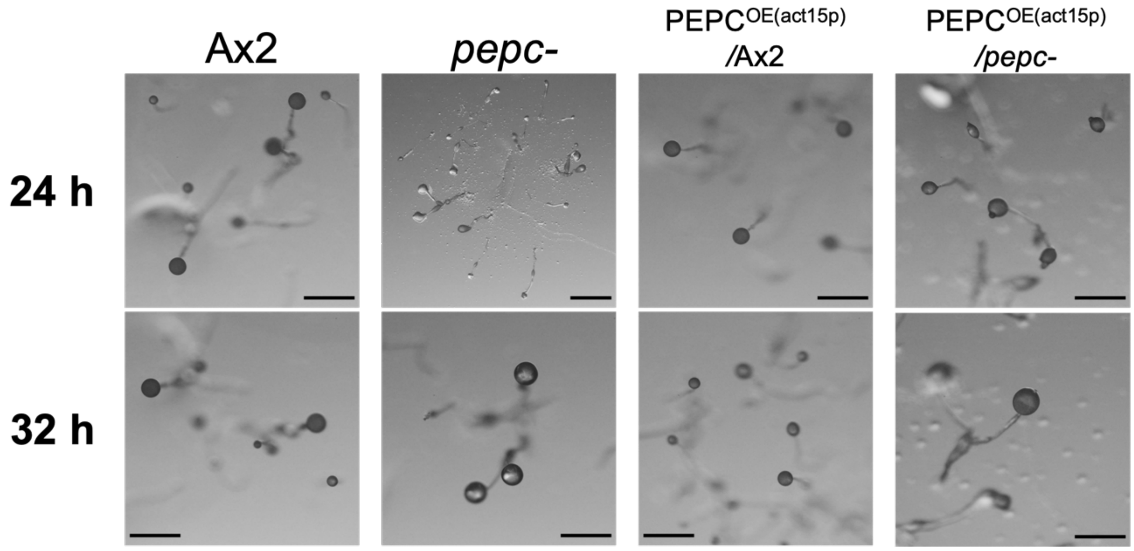

Supplementary Figure 4. Fruiting bodies of Ax2, *pepc-*, PEPC$^{OE(act15p)}$/Ax2 and PEPC$^{OE(act15p)}$/*pepc-* cells developed on water agar plate. Ax2 and *pepc-* cells harboring act15p:PEPC-GFP as PEPC overexpressor. Bar: 0.5 mm

Species	Genbank or UniProt ID
Acanthaamoeba castellanil str. Neff	XP_004339280
Acytostelium subglobosum LB1	XP_012755779
Alkalispirillum mobile	WP_121441900
Arabidopsis thaliana pepc1	Q9MAH0
Arabidopsis thaliana pepc2	Q5GM68
Arabidopsis thaliana pepc3	Q5GM68
Arabidopsis thaliana pepc4	AEE34835
Corynebacterium glutamicum	P12880.4
Dictyostelium discoideum	EAL63508
Dictyostelium purpureum	XP_003287447
Escherichia coli K-12	P00864.1
Glycine max pepc1	BAA03100
Glycine max pepc4	AAS67006
Glycine max pepc7	BAA23419
Glycine max pepc16	BAA01560
Glycine max pepc17	Q6Q2Z9
Methylonatrum kenyense	WP_248568344
Oryza sativa pepc	Q9FYX8
Oryza sativa pepcb	BAD44938
Polychytrium aggregatum	XP_052963561
Polysphondylium violaceum	KAF2070225
Powellomyces hirtus	KAI8914076
Rhodothermus obamensis	Q59757.1
Salmonella enterica subsp. enterica serovar Typhimurium str. LT2	Q8ZKM0.1
Thermostichus vulcanus	BAB64533
Vibrio cholerae	Q9KNT4.2
Yersinia pestis CO92	Q8ZA84.1
Zea mays pepc1	P04711.2
Zea mays pepc2	P51059.1

Supplementally Table 1. GenBank or UniProt ID of PEPCs used in the phylogenetic analysis

Name	5'- Sequences -3'	used for
PEPCp 06f(Xho1)	CGCTCGAGACAAATATTTCTTAAACTTCC	amplification of PEPC promoter
PEPCp 07r (Bgl2)	CCGAGATCTTGCTAAACCCATTTTTAAAAT	amplification of PEPC promoter
PEPCp 06f (NgoMIV)	CGGCCGGCACAAATATTTCTTAAACTTC	amplification of PEPCp:FP to make dual vector
PEPCp 08r (Bgl2)	GCCAGATCTTTTTAAAATGGACCTTTGTGATAAAAT	amplification of PEPC promoter for OE plasmid
pepc 02f CDS for1209 v2	ATAAAAATCAGATCTAAAAATGGGTTTAGCA	amplification of PEPC coding region
pepc 01r CDS noSTOP for1209 v2	TGAACCACCACTAGTACCAGTATTTTTCAT	amplification of PEPC coding region
pepc 02f CDS (Bgl2)	CCGAGATCTTTAAAAATGGGTTTAGCAGGT	amplification of PEPC coding region
PEPC R H154N v3	AATTTGTGTTGGATTTGCCGTTAAAACAAG	amplification of point-mutated PEPC (H154N) fragment
PEPC F H154N	AATCCAACACAAATTATGAGACGTACTATA	amplification of point-mutated PEPC (H154N) fragment
PEPC R H621P	ACCACCTCTACCAGGGAATAAAGTTAATTT	amplification of point-mutated PEPC (H621P) fragment
PEPC F H621P v2	CCCTGGTAGAGGTGGTACAGCTGCAAGAGG	amplification of point-mutated PEPC (H621P) fragment
PEPC R R629S	ATTACCACCACCGCTTGCAGCTGTACCACC	amplification of point-mutated PEPC (R629S) fragment
PEPC F R629S v2	AGCGGTGGTGGTAATTCACATGAGGGTATT	amplification of point-mutated PEPC (R629S) fragment
PEPC_Xba1_CDS_Infusion_1209_F	AATAAAAATCAGATCTCTAGAAAAAAATGGGTTTAGCAGGTAC	amplification of PEPC coding region with Xba1 site
PEPCp-Xba1_rev_Infusion	CCCATTTTTTTCTAGATTTTAAAATGGACCTTTGTG	amplification of PEPC promoter region with Xba1 site
PEPC KO f2 (EcoR1)	GAATTCGGCAATGGGTGAAGCATTA	making of DNA probe for Southern blot
PEPC R for Southern	TCTTGAAAAGTAACTAACTTGTCTGGTAAC	making of DNA probe for Southern blot
pepc-guide_sense	AGCATCTGCTGGTATGAAAAATAC	making of guide RNA inserted in pTM1285
pepc-guide_antisense	AAACGTATTTTTCATACCAGCAGA	making of guide RNA inserted in pTM1285
pepc f forRT	ATGGGTTTAGCAGGAGATTCACCATATGAT	Reverse transcriptase PCR
pepc r forRT	TTGAGAGTGTTAAACCAAATGATTTCAAAC	Reverse transcriptase PCR
ig7 F	TTACATTTATTAGACCCGAAACCAAGCG	Reverse transcriptase PCR
ig7 R	TTCCCTTTAGACCTATGGACCTTAGCG	Reverse transcriptase PCR
act8T r (NgoMIV)	CGGCCGGCTATCTTTTTGATTTTCATACTC	amplification of promoter region, coding sequence and terminator region

Supplementally Table 2. Primer sequences used in this study

Supplemental Movie S1. The movie corresponds to Figure 5A. Time-lapse imaging of Ax2 cell carrying cell-type specific reporters (green: ecmAO:Achilles, magenta: pspA:mCherry). Merged panels also contain transmitted light images in grayscale. Bar: 100µm.

Supplemental Movie S2. The movie corresponds to Figure 5B. Time-lapse imaging of *pepc-* cell carrying cell-type specific reporters (green: ecmAO:Achilles, magenta: pspA:mCherry). Merged panels also contain transmitted light images in grayscale. Bar: 100µm.

Supplemental Movie S3. The movie corresponds to Figure 6A. Time-lapse imaging from streaming to early slug stage of *pepc-* (green: act15p:GFP) and Ax2 (magenta: act15p:mCherry) chimera at the ratio of 1:9. Bar: 200µm.
